# Predicting apparent passive permeability of Caco-2 and MDCK cell-monolayers: A mechanistic model

**DOI:** 10.1371/journal.pone.0190319

**Published:** 2017-12-27

**Authors:** Kai Bittermann, Kai-Uwe Goss

**Affiliations:** 1 Analytical Environmental Chemistry, Helmholtz Centre for Environmental Research - UFZ, Leipzig, Germany; 2 Department of Chemistry, University of Halle-Wittenberg, Halle, Germany; Griffith University, AUSTRALIA

## Abstract

Experimentally derived apparent permeabilities, *P*_app_, through cell monolayers such as Caco-2 and MDCK are considered to be an in-vitro gold standard for assessing the uptake efficiency of drugs. Here, we present a mechanistic model that describes ‘passive’ *P*_app_ values (i.e., neglecting active transport) by accounting for the different resistances solutes encounter when permeating a cell monolayer. We described three parallel permeation pathways, namely a cytosolic-, paracellular-, and lateral route, each of which consists of a number of serial resistances. These resistances were accounted for via a mechanistic depiction of the underlying processes that are largely based on literature work. For the present *P*_app_ dataset, about as much chemicals are dominated by the cytosolic route as were dominated by the paracellular route, while the lateral route was negligible. For the cytosolic route by far the most chemicals found their main resistance in the various water layers and not in the membrane. Although correlations within the subclasses of chemicals dominated by a specific permeation route were rather poor, we could overall satisfyingly predict *P*_app_ for 151 chemicals at a pH of 7.4 (R^2^ = 0.77, RMSE = 0.48). For a specific evaluation of the intrinsic membrane permeability, *P*_m_, a second experimental dataset based on experiments with black lipid membranes, BLM, was evaluated. *P*_m_ could be predicted for 37 chemicals with R^2^ = 0.91 and RMSE = 0.64 log units.

## Introduction

Apparent cell permeabilities, *P*_app_, through monolayers of human intestinal epithelial cells, originated from human colorectal carcinoma cells (Caco-2) and Madin−Darby Canine Kidney cells (MDCK), are widely considered to be the in vitro gold standard for assessing the uptake efficiency of chemicals into the body [1–4]. *P*_app_ values of MDCK cell lines are also used to estimate the effect of the blood-brain barrier (BBB) [5–7]. Given that these tests are time- and cost-intensive [8], a mechanistically based, sound model that predicts *P*_app_ of organic chemicals from their respective molecular structure is appealing. To the best of our knowledge, no such model has yet been presented and validated for a big variety of chemicals, despite much work being published initializing such a model (see, e.g., [4,9,10] and other literature cited in this work). *P*_app_ can be described, based on the solubility diffusion concept [11], as a series of parallel and serial resistances as we will outline in detail below.

This work is focused on ‘passive’ apparent cell permeability, governed by the diffusion along a concentration gradient according to Fick’s law. We do not discuss ‘active’ transport accomplished by any kind of transporter protein (may it be a transporter protein driven energy consuming transport of chemicals against the concentration gradient or a transport by equilibrative transport proteins resulting in concentration equilibration). The transport of chemicals via transporter proteins is potentially also part of *P*_app_ measured with Caco-2/MDCK cell lines (see, e.g., [12]) and can be important for the overall uptake efficiency, but is not covered by the physicochemical parameters used for our predictions. We restrain our model to the prediction of passive permeability as defined above, because passive permeability remains a ubiquitous part of cell permeation and needs to be understood thoroughly, before active transport can reliably be assessed. Hence, the goal of this work is to outline a mechanistic model to describe *P*_app_ (Caco-2/MDCK, pH 7.4) and to apply it to literature data (151 different chemicals) in order to ascertain the influence of the different transport resistances for a cellular monolayer. As part of our validation procedure we also collected and modeled a second, independent dataset of ‘pure (or intrinsic)’ membrane permeabilities, *P*_m_, derived from black lipid membrane (BLM) experiments (see, e.g., [13] and references below). As discussed in detail in this work, we regard the BLM experiments as gold standard for determining *P*_m_ values, because they are free of artifacts that may accompany cell-line experiments such as metabolism, active transport or ion-trapping in lysosomes. Also, within the available BLM datasets much care has been taken to calculate out the influence of stagnant water layers by performing pH dependent measurements.

## Materials and method

### Experimental data compilation

We collected experimental Caco-2 and MDCK cell line apparent permeability data at a pH of 7.4, *P*_app_ (pH 7.4), from the following literature [1,3,6,7,14–20]. This literature selection is based on a recent publication by Alex Avdeef [9], who also reanalyzed many of the herein collected data, albeit with a different focus. Combining the permeability data derived from Caco-2 and MDCK cell monolayers in one dataset is commonly done in the literature but goes along with some uncertainties, which are generally smaller than one order of magnitude [9,14,21,22]. This should be kept in mind when the modelling accuracy is assessed.

Overall, we collected 222 *P*_app_ (MDCK, pH 7.4) values measured at 37°C, of which 109 had efflux ratios reported, and 143 *P*_app_ (Caco-2, pH 7.4) values, of which 75 had efflux ratios reported, for altogether 191 different chemicals. From this compilation we separated out 53 *P*_app_ (MDCK, pH 7.4) values and 12 *P*_app_ (Caco-2, pH 7.4) values which had efflux ratios of 2 or higher. For the remaining data, multiple *P*_app_ (pH 7.4) values reported in one source, including *P*_app_ (pH 7.4) values measured in both directions (apical to basolateral and basolateral to apical), were averaged. These, still cell line specific, ‘passive’ apparent cell permeability data were finally combined: all experimental data for a given chemical (irrespective of the cell line) that had stirring speeds of 150 rounds per minute (rpm) or lower (including those where no stirring speed was reported) were summarized via the arithmetic mean, because differences in the permeability through stagnant water layers for these mildly or unstirred test conditions cannot be distinguished [23]. If the reported stirring speeds were 150 rpm or higher, the respective value had been taken for the modelling of the *UWL* as outlined below. This resulted in the final dataset comprising 159 *P*_app_ (Caco-2/MDCK, pH 7.4) for 151 different chemicals (8 chemicals are listed twice due to different experimental stirring speeds and are modeled separately, as discussed below). All experimental values and model parameters are given in the S1 Supporting Information and can also be provided by the authors in an Excel file including the predictions upon request.

The *P*_app_ values investigated here are defined according to the transport Eq (1) (i.e., Fick’s law of diffusion) and have been determined via the measurement of the flux resulting from a concentration gradient between a well-mixed donor and a well-mixed acceptor compartment:

$$Flux(pH\;7.4)\;=\;-P_{app}(Caco-2/MDCK,\;pH\;7.4)*\;S*(c_{acceptor}-c_{donor})$$ (1)

*S* is the surface area of the filter the cell-monolayers are grown on and *c* the concentration in the compartment denoted in the subscript. Note that *c* stands for the total concentration of all species of a chemical (this is relevant for ionizable chemicals). We note that there are ample possibilities for artifacts in conducting permeability studies with cell monolayers [8] and we are not in a position to rule out all artifacts in the data that we collected.

### Theory and modelling

**Fig 1:**
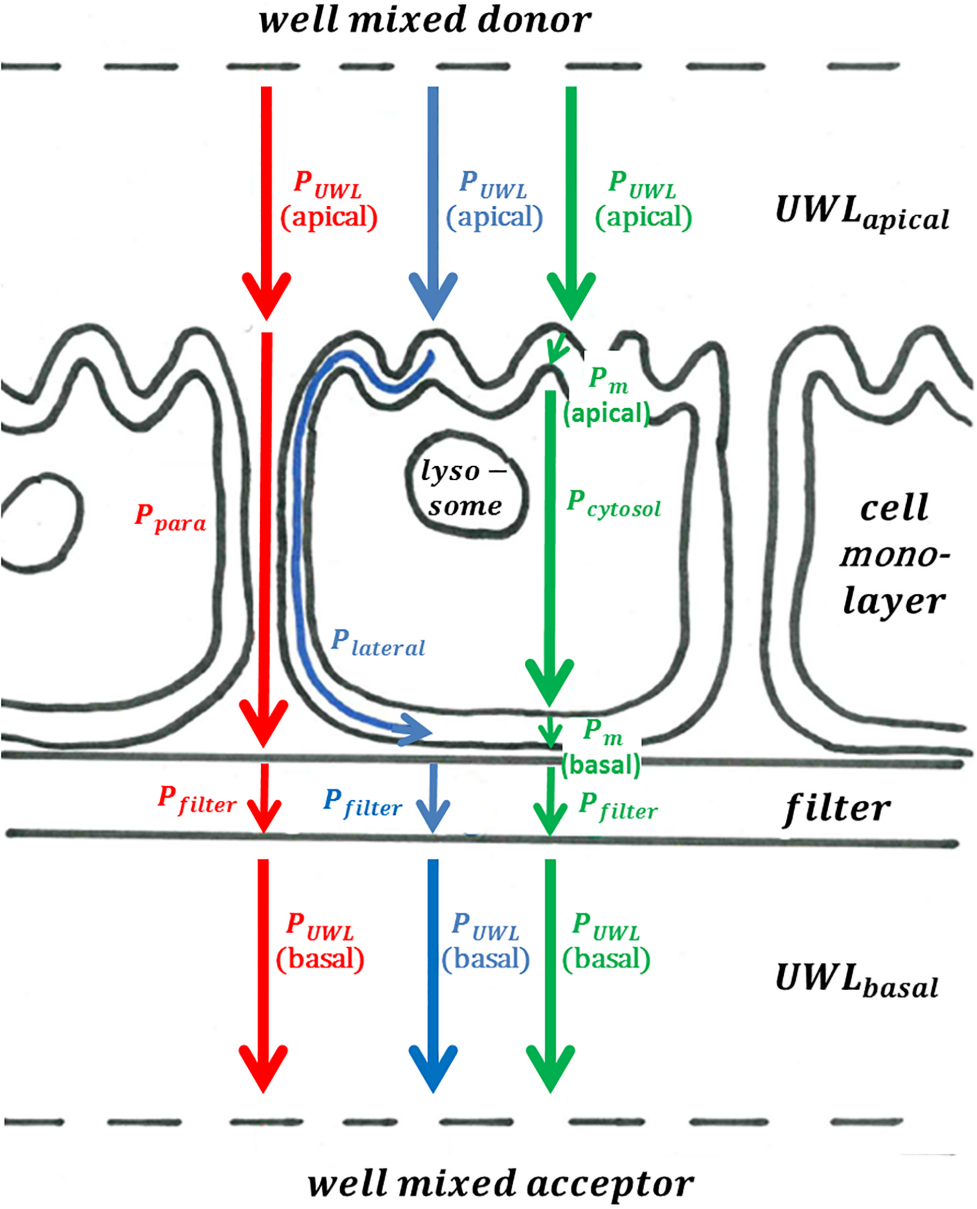
Mechanistic overview showing the three modeled parallel permeability pathways in three different colors (red, blue and green) as well as the serial particular permeabilities they comprise. The paracellular permeation pathway,$P_{para}^{total}$, is shown in red and is comprised of *P*_UWL_(apical), *P*_para_, *P*_filter_ and *P*_UWL_(basal); the lateral permeation pathway, $P_{lateral}^{total}$, is shown in blue and is comprised of *P*_UWL_(apical), *P*_lateral_, *P*_filter_ and *P*_UWL_(basal);, and finally the cytosolic permeation pathway, $P_{cytosol}^{total}$, is shown in green and is comprised of *P*_UWL_(apical), *P*_m_, *P*_cytosol_, *P*_m_, *P*_filter_ and *P*_UWL_(basal).

Adapting the terminology suggested by Heikkinen et al. [24], we differentiate between the resistance originating from the cell membranes and the resistance originating from aqueous boundary layers (*ABL*). Thereby, the *ABL* consist of several serial and parallel resistances (Fig 1) which have to be modeled separately: the unstirred water layer (*UWL*) adjacent to the cell monolayers, the water filled pores of the filter material, the paracellular space and the cytosol. Permeability through the ABL can be a crucial barrier, especially for chemicals with high membrane permeability [25].

For the permeation through a cellular monolayer grown on a filter we assume 3 parallel permeation pathways as outlined in Fig 1, which is a commonly accepted approach (see, e.g., [9,24]). This results in the following equation:

$$P_{app}(Caco-2/MDCK,pH7.4)\;=\;P_{para}^{total}+P_{lateral}^{total}+P_{cytosol}^{total}\;=\;\frac{1}{R_{para}^{total}}+\frac{1}{R_{lateral}^{total}}+\frac{1}{R_{cytosol}^{total}}$$ (2)

Where *P*_app_ (Caco-2/MDCK) is the apparent cell permeability through the Caco-2/MDCK cell monolayer at a pH of 7.4, $P_{para}^{total}$ is the permeability of the paracellular permeation pathway, $P_{lateral}^{total}$ is the permeability of the lateral permeation pathway and $P_{cytosolic}^{total}$ is the permeability of the cytosolic permeation pathway. These three distinct parallel pathways can also be expressed via their respective reciprocal resistances, which are further split up into a number of serial resistances, *R*_*x*_:

$$R_{para}^{total}(pH\;7.4)\;=\;R_{UWL}(apical)+R_{para}(pH\;7.4)+R_{filter}+R_{UWL}(basal)$$ (3)

$$R_{lateral}^{total}(pH\;7.4)\;=\;R_{UWL}(apical)+R_{lateral}(pH\;7.4)+R_{filter}+R_{UWL}(basal)$$ (4)

$$R_{cytosol}^{total}(pH\;7.4)\;=\;R_{UWL}(apical)+R_{m}(apical,\;pH\;7.4)+R_{cytosol}+R_{m}(basal,\;pH\;7.4)+R_{filter}+R_{UWL}(basal)$$ (5)

Modelling of the particular resistances of barriers, *R*_bar_, is discussed one by one in the following subsections. In our model the paracellular resistance, *R*_para_, the lateral resistance, *R*_lateral_, and the membrane resistance, *R*_m_, are estimated for the external pH of 7.4. According to the pH partition hypothesis [9], we assume that only the neutral form of the respective chemical can pass the latter two resistances, *R*_lateral_ and *R*_m_. Hence, chemicals that are mostly ionized at pH 7.4 and therefore pass all *ABL* resistances mostly in the ionized form will have to undergo an acid-base reaction in the transition region between water and the membrane before they can pass the membrane as a neutral species. In line with the pH partition hypothesis we assume here that this acid-base reaction occurs instantaneously and is not kinetically hindered (see below for further discussion). For the speciation calculations we use a pH of 7.4 for all aqueous compartments including the cytosol according to the literature [26,27].

For the sake of brevity, the calculation of all thermodynamic coefficients used for the modelling is described in the S1 Supporting Information, where we also give a list of all abbreviations.

### Solubility diffusion model

The basis for the description of all the particular resistances outlined below is the solubility diffusion model, comprehensively discussed, e.g., in [11,28] and only shortly revisited here. Imagine two well mixed water-compartments, which are separated by a homogeneous barrier, indicated by the index ‘*bar*’, which could, e.g., be some kind of membrane but also a stagnant water layer of a certain thickness or a porous material with limited diffusional space. Now a solute is introduced in the donor compartment. Assuming homogeneous concentrations throughout the donor and acceptor compartment, the resulting concentration gradient in the barrier at steady state conditions will be linear and the flux can thus, according to Fick’s law, be described as

$$Flux\;=\;\frac{-D_{bar}K_{barw}S_{bar}*(c_{acceptor}-c_{donor})}{h_{bar}}$$ (6)

Where *D*_bar_ is the diffusion coefficient in the barrier, *K*_barw_ is the partition coefficient between the barrier and water (the higher the partitioning from water into the barrier, the more molecules can diffuse through the barrier at any moment in time at an identical *D*_bar_), *S*_bar_ is the area across which diffusional transport occurs, c_x_ stands for the respective aqueous concentrations and *h*_bar_ is the thickness of the barrier, i.e., the distance that has to be overcome by diffusion. In order to describe the intrinsic resistance of a barrier, *R*_bar_, which is reciprocal to its permeability, *P*_bar_, the parameters *D*_bar_, *K*_barw_ and *h*_bar_ can be lumped together as follows:

$$R_{bar}\;=\;\frac{1}{P_{bar}}\;=\;\frac{h_{bar}\;\varepsilon_{bar}}{D_{bar}K_{barw}}$$ (7)

*ε*_bar_ can be interpreted as a porosity which is needed in Eq 7 if the actual area through which diffusion can occur is smaller than the area *S*_bar_ (i.e., *ε*_bar_ = actual diffusive area/*S*_bar_). This Eq (7) is the basis of our description of all particular resistances outlined in Fig 1, even if the specific expressions may eventually look quite different. For the stagnant, unstirred water layer, *UWL*, the partition coefficient *K* between the well-mixed water compartment and the *UWL* equals unity (at steady state) for all solutes, so that in this case the aqueous diffusion coefficient, *D*_aq_, and the thickness of the UWL, *h*_UWL_, dominate the overall resistance of the UWL, *R*_UWL_. For the membrane, however, *K* has a much stronger influence (as it varies over several orders of magnitude for different solutes) than *D* (which differs by less than a factor of 4 in our dataset)–while *h* is only a constant factor (albeit much smaller for a membrane as compared to the *UWL*).

According to the pH partition hypothesis we assumed the permeation through the water-filled compartments, i.e., the *UWL*, cytosol and filter to be independent of the speciation, i.e., neutral and ionic species permeate with the same velocity. In contrast, the permeation through the membrane, lateral and perpendicular to the membrane plane, was only attributed to the fraction of the respective neutral species. In the special case of the paracellular permeation we took all different pH dependent microspecies into account and the electric field acting on them (see below).

### Permeability through the unstirred water layer, *P*_*UWL*_

The UWL is a poorly stirred region of water, where the diffusional movement of molecules exceeds the convectional movement [25,29]. The thickness of the UWL, *h*_UWL_, depends nonlinearly on the stirring speed. Analogously to the procedure outlined elsewhere [23], we calculated the UWL resistance, being the reciprocal UWL permeability, via the stirring speed using a hydrodynamic model of the form

$$R_{UWL}\;=\;1/P_{UWL}\;=\;R_{UWL}(apical)+R_{UWL}(basal)\;=\;1/\;({Χ}_{UWL}*v^{\alpha })$$ (8)

*Χ*_*UWL*_ is a constant derived from the aqueous diffusivity of a typical solute to the power of 2/3, the kinematic viscosity (to the power of -1/6) and geometrical factors of the permeation cell. Reported values for *Χ*_*UWL*_ range from 0.57*10^-6 cm/s [10] to 4.1*10^-6 cm/s [30]; *ν* is the stirring speed (in rpm) and *α* is an empirical constant with values reported ranging from 0.8 [30] to 1 [10] (theoretically, α is expected to be 0.5 [31]). In our model we used a *Χ*_*UWL*_ value of 2*10^-6 cm/s and an α value of 0.6, based on a refit of the literature data [9,10]. In those cases where no stirring was done at all in the experiment (or when no stirring was reported), or the reported stirring speed was below 150 rpm, we took the default value of 150 rpm. As shown by Avdeef et al. [23], the differences in *P*_*UWL*_ arising from variable stirring conditions below 150 rpm cannot reliably be distinguished. Korjamo et al. concluded that pH dependent measurements are generally better suited to derive the *UWL* thickness (and thus *P*_*UWL*_) than experiments with different stirring speeds [29], as it was also shown by Avdeef et al. for a small set of chemicals [23]. However, in a purely predictive model there is no other way then estimating *P*_*UWL*_ via the stirring speed. Besides, the *UWL* is in reality even more difficult to grasp: the thickness of the *UWL* is also dependent on the size and diffusion coefficient of the investigated chemical [32,33]. But this attention to detail is beyond the scope of this work.

### Paracellular permeability, *P*_*para*_

For the calculation of the paracellular resistance we used the approach outlined in references [21,31], which accounts for the porosity ε_para_ and the tortuous path length δ, the size restricted diffusion and the influence of an electric field *E* with a potential drop Δφ along these pores:

$$R_{para}\;=\;1/P_{para}\;=\;{\left\{\frac{\varepsilon_{para}}{\delta }D_{aq}(37{}^{\circ}C)F(\frac{r_{hydr}}{r_{pore}})E(\Delta \varphi )+\frac{\varepsilon_{para}}{\delta_{2}}D_{aq}(37{}^{\circ}C)\right\}}^{-1}$$ (9)

Our parametrization is as follows: *ε*_*para*_*/δ* = 0.78, Δφ = 30mV and the pore radius *r*_pore_ of 12.9 Å was taken from references [31,34] and represents typical values according to Table 8.2 in reference [31]. For *ε*_*para*_/*δ*_2_, describing the secondary pore population, we used the value of 0.05 in accordance with the condition *ε*_*para*_/*δ* > *ε*_*para*_/*δ*_2_ as stated in [21]. We note that for the calculation of the Renkin hydrodynamic sieving -function for cylindrical water channels *$F(\frac{r_{hydr}}{r_{pore}}\}$* (see Eq. 8.7 to 8.9 in reference [31]) it is the dynamic viscosity, ƞ (being 0.6913E-3 N*sec/m2 at 37°C), and not the kinematic viscosity of water as stated by Avdeef that has to be used for deriving the hydrodynamic radius *r*_hydr_:

$$F(\frac{r_{hydr}}{r_{pore}})\;=\;{\left(1-\frac{r_{hydr}}{r_{pore}}\right)}^{2}*\left\{1-2.104(\frac{r_{hydr}}{r_{pore}})+2.09{\left(\frac{r_{hydr}}{r_{pore}}\right)}^{3}-0.95{\left(\frac{r_{hydr}}{r_{pore}}\right)}^{5}\right\}$$ (10)

where the molecular hydrodynamic radius, *r*_hydr_, is calculated via a combination of the Sutherland-Stokes-Einstein spherical particle equation and the Stokes-Einstein equation:

$$r_{hydr}\;=\;\left(0.92+\frac{21.8}{MW}\right)\frac{k_{B}T}{6\pi ƞD_{aq}(37{}^{\circ}C)}10^{8}$$ (11)

where *k*_B_ is the Boltzmann constant, *T* is the absolute temperature (310 K), *MW* is the molecular weight and *D*_aq_ the aqueous diffusion coefficient.

For the influence of the electric field we assumed that zwitterions are not affected and are thus treated like neutral species, while divalent anions and divalent cations are affected by the electric field (which is due to negatively charged residues along the pore channel) twice as much as their monovalent counterparts:

$$E(\Delta \varphi )\;=\;f_{neutral}+f_{zwitterion}+f_{cation}\frac{\kappa |\Delta \varphi |}{1-e^{-\kappa |\Delta \varphi |}}+f_{anion}\frac{\kappa |\Delta \varphi |}{e^{\kappa |\Delta \varphi |}-1}+f_{dication}\frac{2\kappa |\Delta \varphi |}{1-e^{-2\kappa |\Delta \varphi |}}+f_{dianion}\frac{2\kappa |\Delta \varphi |}{e^{2\kappa |\Delta \varphi |}-1}$$ (12)

with *f*_x_ being the fraction of the chemicals that exists as species *x* (being neutral, anionic, cationic, dianionic, dicationic or zwitterionic) at the experimental pH of 7.4 and κ being a constant of 0.037414mV^-1^ at 37°C (κ = F/N_A_ *k*_B_T, where F is the Faraday constant and N_A_ Avogadro’s number).

### Permeability through the cytosol, *P*_*cytosol*_

We modeled the cytosol according to Verkman [35] as a crowded, watery compartment with diffusion coefficients in the cytosol being a quarter of the respective diffusion coefficients in pure water (the ratio was experimentally found for the fluorescent molecule BCECF (molecular weight (*MW*) 880.75)). This assumption seems reasonable, as 70% of the cytosol consists of water [36] with the rest being mostly large macromolecules hampering the diffusion of solutes due to collision [27]. Approximating the partition coefficient between water and cytosolic water as being roughly equal to one (note that the partition coefficient between the different immobile cytosolic compartments, e.g., macromolecules like proteins, and water can be very different from unity—but this does not matter for the steady state flux we are aiming to describe in our model), we therefore modeled the cytosol resistance, *R*_cytosol_, via

$$R_{cytosol}\;=\;\frac{h_{cytosol}}{0.25*D_{aq}(37{}^{\circ}C)}$$ (13)

For the determination of the thickness of the cytosol, *h*_cytosol_, we assumed the cells to be spherical. Averaging the cell volumes of 2.08 pL for MDCK and 1.27 pL for Caco-2 [37], we determined *h*_cytosol_ to be about 15 μm. The cells obviously deviate from a perfect spherical shape, but the systematic error that goes along with this simplification is within the inevitable errors that occur when lumping MDCK and Caco-2 data together.

### Permeability through the filter, *P*_*filter*_

Porous polycarbonate filters serve as the supporting material upon which the cell lines are grown. We described the resistance exerted by the stagnant water-filled pores according to the procedure applied in references [9,10]:

$$R_{filter}\;=\;\frac{h_{filter}}{\varepsilon_{filter}D_{aq}(37{}^{\circ}C)}$$ (14)

Here, *h*_filter_ is the filter thickness, i.e., the pore length (10 μm), and *ε*_filter_ is the filter porosity. We used an *ε*_filter_ value of 0.13, which is considered a ‘translucent filter’ (*ε*_filter_ from 0.13 to 0.20), while ‘clear filters’ have an *ε*_filter_ value of 0.05 [9].

### Lateral permeability along the membrane, *P*_*lateral*_

Consistent with our description of the permeation through the cytosol, we assumed the cells to be spherical for the description of the lateral permeation pathway, i.e., the diffusion of solutes along the membrane plane. We calculated *R*_lateral_ with the following formula:

$$R_{lateral}(pH\;7.4)\;=\;\frac{h_{lateral}*\phi }{f_{neutral}(pH\;7.4)*K_{lipw}*D_{lateral}}$$ (15)

With *h*_lateral_ being the length of the lateral diffusion pathway, ϕ being a factor accounting for the limitation of space available for lateral diffusion, *K*_lipw_ being the liposome-water partition coefficient (which is taken as a surrogate for the membrane-water partition coefficient for real biological membranes [38]) of the neutral fraction of the investigated solute, *D*_lateral_ being the lateral membrane diffusion coefficient further described below and finally *f*_neutral_ being the fraction of the chemical that exists as neutral species at the experimental pH of 7.4 (note that it is a simplification assuming that only the neutral fraction is permeating laterally, as also ions do partition in phospholipid membranes, see, e.g. [39]). *K*_lipw_ is only used here in the description of *R*_lateral_ because the molecules will move laterally in those parts of the membrane that they prefer. *K*_lipw_ is a parameter that is averaged over the whole (membrane) phase and is actually dominated by those parts of the membrane that are preferred by the solutes; and those parts of the membrane preferred by the solutes are just not those parts that dominate the transport resistance. Polar molecules e.g., might sorb strongly into the zwitterionic membrane headgroup region, which results in a high *K*_lipw_ [40] and might make the lateral permeation route attractive to those chemicals, while the same polar molecules are unlikely to permeate the phospholipid membranes due to unfavorable interactions with the alkane-like membrane interior, resulting in a high membrane resistance for those solutes (which can be described via the *K*_hexw_ as outlined below and Fig 1 in S1 Supporting Information). The factor ϕ is the ratio between the space available for the solute to sorb into the membrane and the space available for the solute to diffuse on the lateral pathway parallel to the cytosolic or the paracellular pathway. This restricted diffusional space is equivalent to the porosity, *ε*_filter_, introduced above to describe *R*_filter_, and becomes obvious when looking at the monolayer from the top view (see Fig 3 (b) in Hubatsch et al. [8], where the cell borders are stained green, which corresponds to ϕ):

$$\phi \;=\;\left(\frac{\pi {\left(0.5*h_{cytosol}\right)}^{2}}{\pi {\left(0.5*h_{cytosol}+h_{membrane}\right)}^{2}-\pi {\left(0.5*h_{cytosol}\right)}^{2}}\right)≅682$$ (16)

The factor ϕ is only a rough estimation and should account for the fact that all the space taken up by the cytosol is not available for the lateral diffusion pathway. The height of the membrane, *h*_m_, is approximately 55 Å. As conceivable from Fig 1, the length of the lateral diffusion pathway, *h*_lateral_, depends on where the solute sorbs to the membrane on the apical side and where it desorbs on the basal side. Here we assumed the starting and endpoint of the lateral diffusion to be at the respective ‘center’ of the apical and basolateral side (which is furthest away from the neighboring cells). Hence, *h*_lateral_ was described as

$$h_{lateral}\;=\;0.5*\pi h_{cytosol}≅24\mu m$$ (17)

### Permeability through the membrane, *P*_*m*_

#### Black lipid membrane data

The pharmacologist Charles Ernest Overton reasoned a century ago what is now known as the ‘Overton Rule’: the greater the lipid solubility of a chemical, the greater is the rate of penetration through the plasma membrane [9]. In principle, this is consistent with the more quantitative approach that is known as the solubility-diffusion model [11]. However, in Overton’s concept the phospholipid membrane is regarded as a homogeneous matrix acting as barrier for solute diffusion. This is contrary to the modern day conception of the membrane as a matrix which is highly anisotropic perpendicular to the membrane plane (see, e.g., Bittermann et al. [39]). In other words: while partitioning into a membrane is dominated by those parts of the membrane that are most attractive for a solute, permeability through the membrane is dominated by those parts that are the least attractive. It is therefore no surprise that pure membrane permeability does not correlate well with membrane partition coefficients (see Fig 1 in S1 Supporting Information) or the octanol-water partition coefficient [13]–although the latter correlates strongly with the partitioning of neutral compounds into phospholipid membranes [38]. Here we argue that the dominating resistance within a membrane will usually be located in the non-polar inner part with its long alkyl chains because polar organic chemicals will find this region rather unattractive. For non-polar organic chemicals this approach would obviously not hold, but for these chemicals it can be expected that the dominating resistance is in the *ABL*, so that a correct prediction of *P*_m_ for non-polar chemicals is not crucial in a *P*_app_ model anyway. For ionizable chemicals the situation is less clear. The prevalent ionic species may easily pass the *ABL* and then transform into the corresponding neutral species at the membrane surface. As the neutral form of an acid or a base is not a completely non-polar molecule, we assume that in this case the main membrane resistance is still constituted by the non-polar inner membrane part.

Thus we mimic the overall membrane resistance by accounting only for the membrane hexadecane-like interior with a thickness, *h*_hex.-like_, of 15 Å (see Fig 5 in Bittermann et al. [40]). Applying the homogeneous solubility-diffusion model again to this non-polar interior of the membrane, the membrane resistance, *R*_m_, results from

$$R_{m}\;=\;\frac{1}{P_{m}}\;=\;\frac{h_{hex.-like}}{K_{hexw}D_{hex}}$$ (18)

with *D*_hex_ being the diffusion coefficient in hexadecane and *K*_hexw_ being the hexadecane water partition coefficient. In order to test how well the pure bulk phase of hexadecane as a model for the membrane interior predicts the overall membrane resistance, we compiled a black lipid membrane (BLM) permeation dataset from the following literature [33,41–54]. The BLM data only represent the intrinsic permeability of the neutral species of these chemicals because the influence of the *UWL*, where both species still play a role, has been filtered out by measuring the pH dependence of the total permeability [13]. A detailed summary of this experimental dataset is given in the S1 Supporting Information. BLM data have the advantage that they are not affected by paracellular transport, lateral transport or active transport and they are also not subject to artifacts that may arise from metabolism or strong retention in lysosomes. Moreover, in all *R*_m_ data used here, the influence of the *UWL* was already corrected for, based on the pH dependence of the experimental results. The only artifact that one has to be aware of is the possibility of a limited proton flux rate between the well mixed water compartment and the membrane surface which would limit the acid-base reactions that occur in the transition region between the *UWL* and the membrane for dissociating chemicals. Gutknecht and coworkers [50] were the first to describe this effect and so one can safely assume that BLM data reported by them as intrinsic membrane permeabilities are not subject to this artifact. For other BLM data such as those from Xiang et al. [48], we were able to rule out such effects in the experimental data by using the mathematical model presented by Antonenko et al. [33] for the respective experimental conditions. Thus, the assembled BLM data are ideally suited to validate our approach for modeling *R*_m_. It has been claimed that experiments using the parallel artificial membrane permeability assay, PAMPA, has equal advantages as the BLM data [23]. However, the experimental PAMPA model does not exhibit a single, real membrane bilayer structure (see, e.g., Fig 7 in reference [55]). In contrast, the BLM experiments do investigate the permeability through a real phospholipid bilayer structure of a well-defined size, which is obviously a crucial precondition to ascertain a mechanistic understanding of *P*_m_.

**Fig 2:**
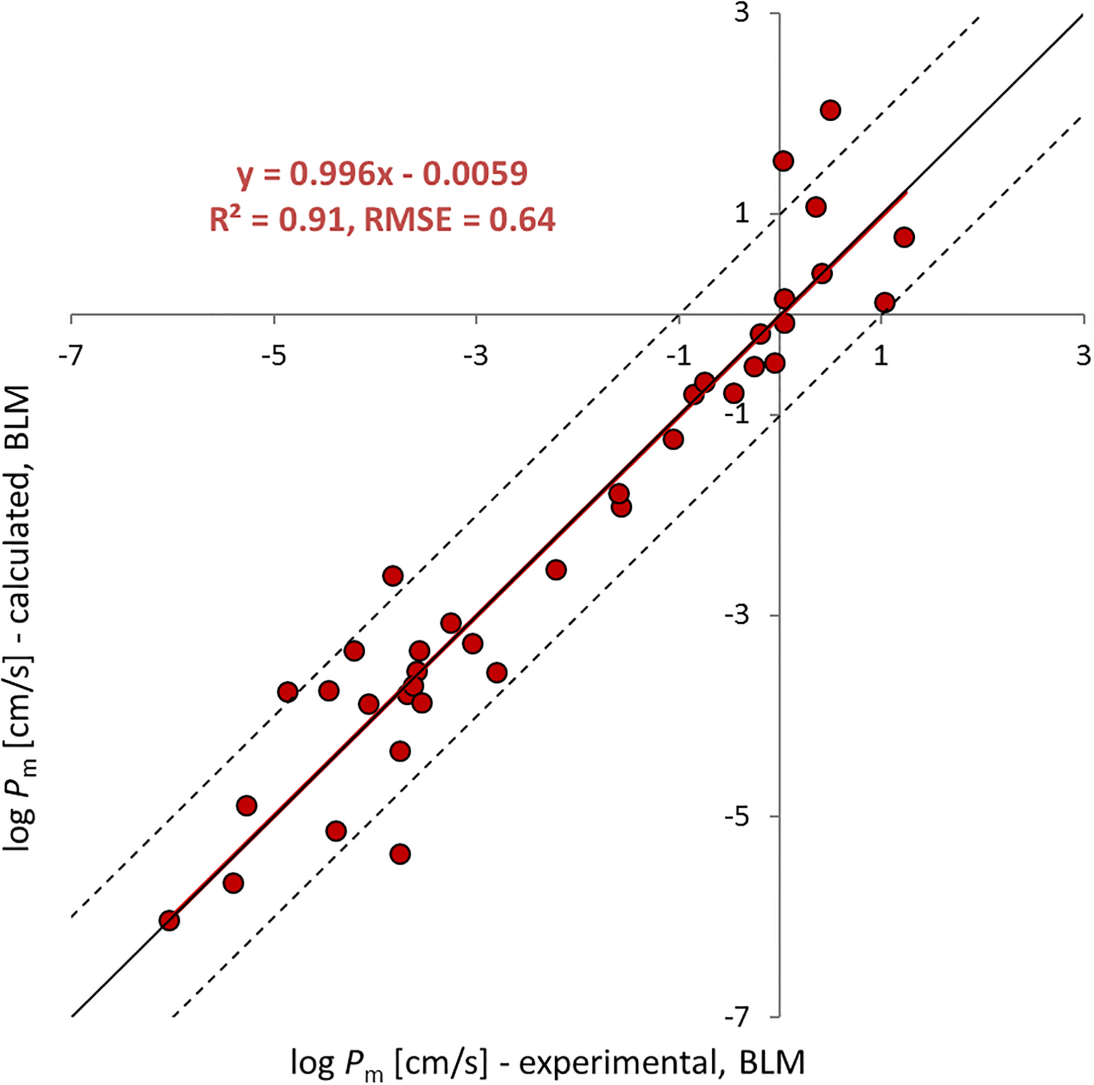
Prediction of BLM permeability with Eq (18) vs experimental values. As discussed in [13], the permeability of small molecules with molecular weights below 50 is higher than expected when modeled with the homogeneous solubility diffusion model. This has been explained by a polymer-like behavior of the membrane that differs from the liquid hydrocarbon-like behavior, which is implicitly presumed in the model. Hence, we excluded the nine smallest molecules with molecular weights <50 (see SI for all values).

A detailed discussion of the data presented in Fig 2 will follow in another paper; for the scope of this work it is important to note that Eq (18) seems to work well despite the inherent simplifications which are also further discussed below. The 47 permeability values from the literature for the neutral species of 37 chemicals span from log *P*_m_ of -6.03 to 1.23 [cm/sec] and are predicted with R^2^ = 0.91, RMSE = 0.64 log units.

#### R_m_ for Caco-2/MDCK data

In our description of *R*_m_ (basal, pH 7.4) we follow the model outlined for BLMs above:

$$R_{m}(basal,pH\;7.4)\;=\;\frac{h_{hex.-like}}{f_{neutral}(pH\;7.4)*K_{hexw}D_{hex}}$$ (19)

For the apical membrane we take into account that the microvilli increase the surface area by a factor of 24 [56]:

$$R_{m}(apical,pH\;7.4)\;=\;\frac{h_{hex.-like}}{24*f_{neutral}(pH\;7.4)*K_{hexw}D_{hex}}$$ (20)

A more in depth discussion of the effect of microvilli can be found in reference [4] (and analogously for macrovilli in reference [57]). It reveals that the increase of surface area is only improving the permeability for those chemicals whose main resistance is located in the membrane and not in the *ABL* (i.e., polar chemicals). Non-polar chemicals that easily pass the membrane actually do not profit from the existence of microvilli so that for them the acceleration factor of 24 should not be applied. However, as before, the overall calculation of *P*_app_ for non-polar chemicals is largely dominated by the *ABL* anyhow so that it does not matter whether *P*_m_ for these chemicals is overestimated or not.

Note that for zwitterionic chemicals, whose net charge is zero, we assumed *P*_m_ to be negligibly small based on the earlier finding that the permeability of zwitterionic amino acids through large unilamellar vesicles composed of egg-PC and DMPC is very low and similar to those of sodium and potassium (in the range of 10^−12^ to 10^−14^ cm/s) [58]. In contrast, Avdeef tacitly treats zwitterionic chemicals equivalent to neutral ones when he extracts *P*_m_ data from experimental *P*_app_ (Caco-2/MDCK) [9].

## Results and discussion

### Prediction quality of estimated *P*_*app*_

The experimental log *P*_app_ (Caco-2/MDCK, pH 7.4) values range from -3.65 to -7.49 cm/s (median -4.94); values from different sources collected for the same chemical differ up to 1.83 log units (median 0.57), while values collected for the same chemical from the same source differ up to 1.31 log units (median 0.31). Note that these reported error margins reflect both systematic errors as well as the inevitable uncertainties arising when merging experimental Caco-2 and MDCK data.

**Fig 3:**
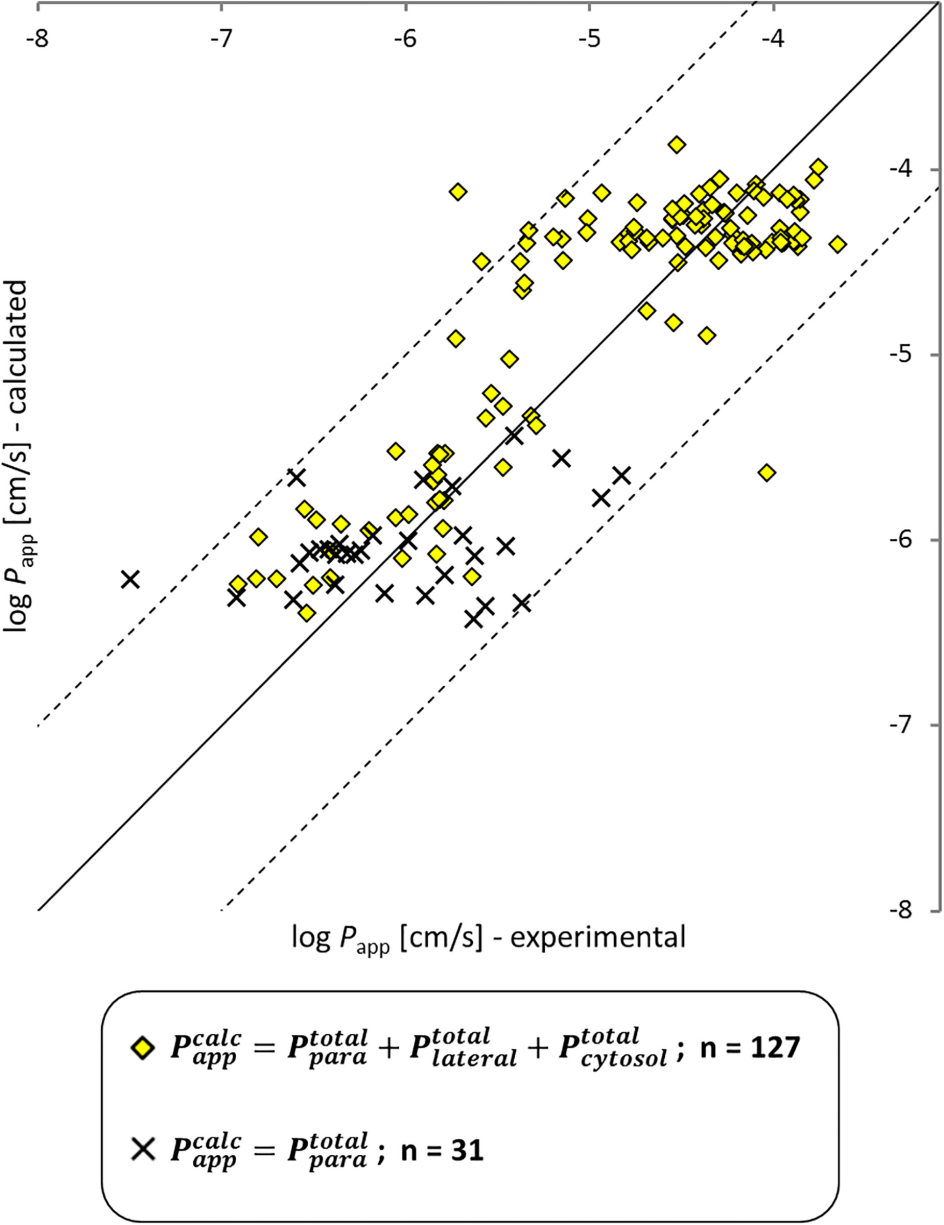
Modeled *vs* experimental apparent Caco-2 and MDCK cell permeability at pH 7.4. Yellow diamonds were modeled with three optional permeation routes (paracellular, cytosolic and lateral), while x-shaped black crosses are chemicals that were a priori limited to the paracellular route, because they were estimated to be 100% ionized. The model has an overall predictive quality of RMSE = 0.48, R^2^ = 0.77.

The model seems to capture the most important permeability features. The prediction of log *P*_app_ (Caco-2/MDCK, pH 7.4) with Eq (2) is satisfying (R^2^ = 0.77, RMSE = 0.48), especially if one considers the high experimental uncertainty that becomes visible in the experimental scatter for single chemicals. Note that also some of the partitioning and diffusion parameters are modeled at different temperatures, which leads to additional uncertainty but is unavoidable when one wants to rely on well calibrated literature models. For two chemicals, predicted and experimental values clearly differ by more than one order of magnitude. Quercetin as one of them (log *P*_app_ (exp) = -4.03, log *P*_app_ (calc) = -5.64) is predicted to possess 17 different relevant ionic species. In this case it is quite likely that the predicted speciation underlying our model is not correct and we therefore ignore this chemical for the remaining discussion. The other one is terfenadine (log *P*_app_ (exp) = -5.71, log *P*_app_ (calc) = -4.12) for which no problem is directly obvious. Note that both $P_{lateral}^{total}$ and $P_{cytosol}^{total}$ from Eq (2) become zero when no neutral fraction is predicted to be present at pH 7.4 (which is the case for the permanently charged bretylium cation and 30 additional chemicals, shown as x-shaped black crosses in Fig 3). The software JChem reports the percentage of neutral fraction only with two decimal places; i.e., if the neutral fraction of an ionizable chemical is smaller than 0.0001 (i.e., 0.01%), the molecule is regarded as fully ionized. For these chemicals our model allows permeation through the cell layer only via the paracellular route, $P_{para}^{total}$, (which is a simplification owing to the accuracy-limits of the state-of-the-art p*K*_a_ prediction methods).

### Main transport resistance

**Fig 4:**
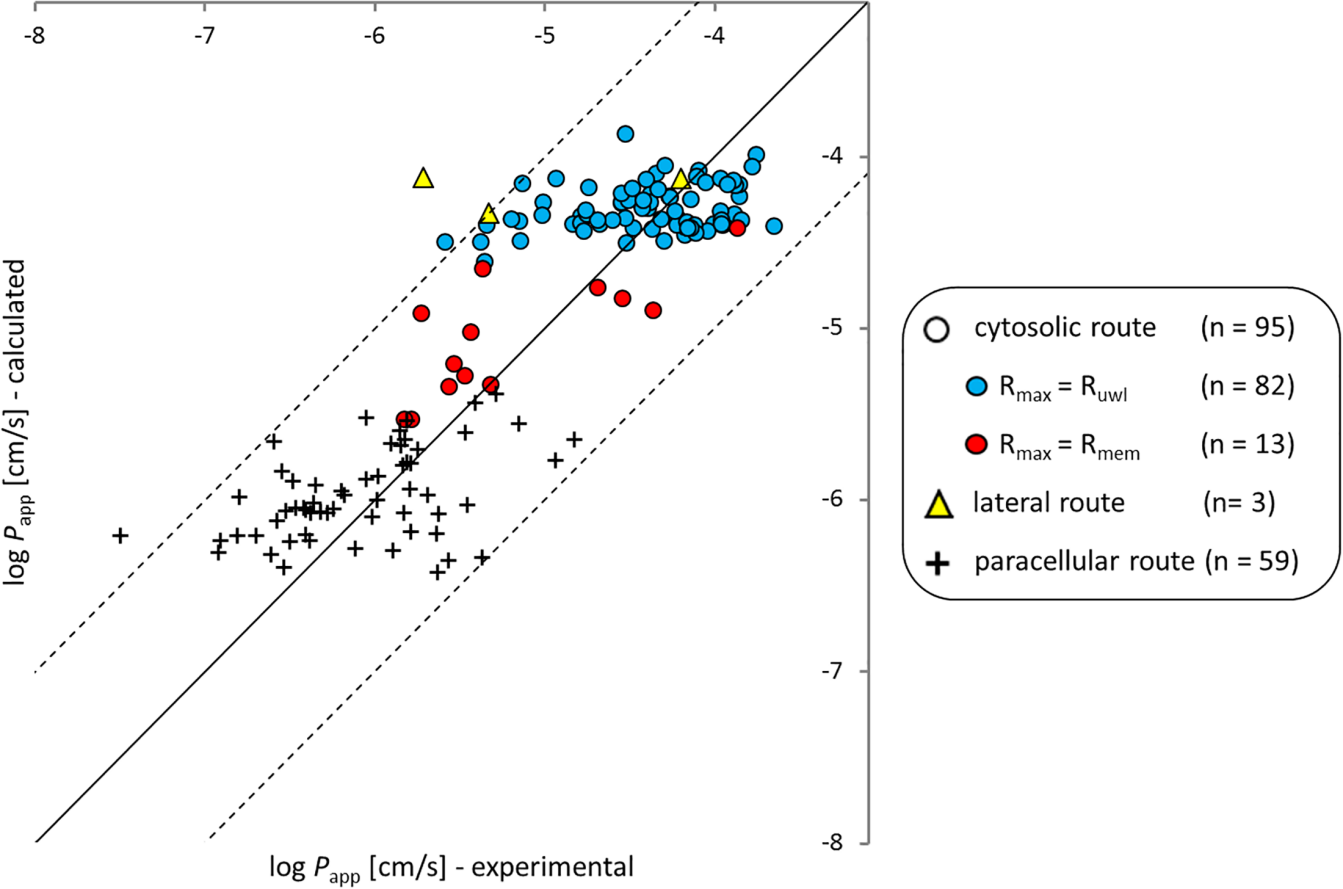
Experimental apparent Caco-2 and MDCK cell permeabilities (pH 7.4) vs calculated values, differentiated according to the most permeable route (i.e., the route with the lowest resistance). The symbol form is according to the permeation route which has the highest permeability as shown in the legend (a circle for the cytosolic route, a triangle for the lateral route, and a plus for the paracellular route. For the 95 chemicals which permeate the cell monolayer predominantly via the cytosolic route, the main resistance within the cytosolic route is color-coded as follows: for the blue circles the UWL is the main resistance, while for red circles the sum of the two membranes (apical plus basolateral) is the main resistance. Quercetin has been removed as discussed above.

According to our model, the cytosolic route and the paracellular route are dominating the overall apparent permeability for most chemicals, while the lateral route hardly plays a role:

When the permeability on the cytosolic route rises over 10^−6^ cm/sec, it starts to dominate over the paracellular route. Within the chemicals whose *P*_app_ is dominated by the cytosolic route (all circles in Fig 4; n = 95) most chemicals (blue circles in Fig 4, n = 82) are restricted by the *UWL* resistance which confines *P*_app_ of all chemicals to about 10^−4^ cm/sec even under strong agitation. Out of the 13 chemicals dominated by *P*_m_ rather than *P*_UWL_ (red circles in Fig 4) most are highly ionized at the pH of 7.4. The remaining chemicals are neutral but very polar (e.g., amiloride, hydrocortisone, methylprednisolone, urea). The predicted log *K*_hexw_ of these 13 chemicals span from -6.18 to -2.23 (median = -4.43). All *P*_app_ values that are dominated by *P*_m_ (red circles in Fig 4) are well predicted and deviate less than one log unit from the identity line (RMSE = 0.42, R^2^ = 0.59) which can be seen as further confirmation of our model concept for *P*_m_, despite the fact that we used a very simplistic approach when we assumed that the main resistance in the membrane stems from a hexadecane-like inner layer. Other researchers have reported quite complex results that seem incompatible with this simplistic approach. For example, the membrane permeability for water, ammonia, glycerol, urea, formamide and acetamide has been investigated by Hill and Zeidel [59], who found a factor of 18–90 difference between both membrane bilayer leaflets of MDCK membranes. The experiments were conducted both with liposomes, mimicking the exofacial lipid leaflet (made of phosphatidylcholine, sphingomyelin, glycosphingolipids and cholesterol) and with liposomes, mimicking the cytoplasmic leaflets (made of phosphatidylethanolamine, phosphatidylserine and cholesterol). It remains unclear whether or not the Caco-2 cells exhibit the same differences between exofacial and cytoplasmic bilayer leaflets, but overall the permeation characteristics between the two cell types are similar [14,21,22]. The resistance of both bilayer leaflets behave independent and additive as was shown via a BLM experiment that reconstructed the asymmetrical MDCK membrane [60]. A good overview of the composition of major lipid components of the two halves of different types of membranes (e.g., human, rat, influenza virus) is given in Jain et al. [61]).

While all this complexity doesn´t seem to hamper our ability to predict *P*_app_ with a simple approach, it must be pointed out that the success in predicting *P*_app_ values comes mostly from a correct classification of the chemicals in terms of their major permeation pathway as shown in Fig 4. Within the classes of *UWL* or paracellular dominated chemicals the predictions do not explain much of the reported experimental variability. For those chemicals that find their major resistance in the *UWL* this is due to our insufficient ability to predict the thickness of the *UWL* in Caco-2/MDCK assays that may have quite different and unknown geometries and for which often no information on agitation is available. This situation could easily be improved in the future if Caco-2/MDCK experiments were better standardized. As soon as the *UWL* thickness is known, it should become straightforward to predict the *UWL* permeability quite accurately from this thickness and the aqueous diffusivity of the solute. For chemicals that permeate via the paracellular pathway the predictions do also not explain the observed scatter (plus signs in Fig 4). In part this might be the result of erroneously assuming (based on the speciation results of JChem) that a chemical occurs as a permanent ion while in reality this chemical has a (small) fraction of neutral species that prefers the cytosolic pathway and thus dominates the overall permeability. The few chemicals that permeate on the cytosolic route and that are dominated by the intrinsic membrane resistance (red circles in Fig 4) do not allow much of a conclusion towards our ability to correctly predict the variability of data for this class of chemicals. However, for this class of chemicals we have our second data set of BLM data that clearly suggests that our submodel for predicting intrinsic membrane permeabilities performs well over a pretty wide range of chemicals and permeabilities (see Fig 2).

### Permeability through the lateral route, *P*_*lateral*_

To the best of our knowledge no focus has been set in the literature on the possible cell permeation via the lateral route (see Fig 1). In our model, *P*_app_ of only three chemicals is predicted to be dominated via the lateral route, as outlined in Table 1.

**Table 1:** List of chemicals for which the lateral route is predicted to have the highest permeability at pH 7.4.

chemical	$log\;R_{para}^{total}$	$log\;R_{cytosol}^{total}$	${log\;R}_{lateral}^{total}$	log *P*_app_ CALC	log *P*_app_ EXP
Saquinavir (+ Ritonavir inhibitor)	6.19	4.79	4.53	-4.33	-5.33
Terfenadine	5.97	4.43	4.43	-4.12	-5.71
Ritonavir	6.34	4.44	4.42	-4.13	-4.20

For all three chemicals listed in Table 1, the cytosolic pathway has to be considered as equally relevant within the model uncertainties. We might have underrated lateral diffusion by not taking the ionic species into account. However, we would argue that almost all of the organic ions will preferably sorb into the zwitterionic headgroup region of the phospholipid membrane bilayer [40] and this region is more densely packed than the inner membrane [62]. Therefore the lateral diffusion coefficient, *D*_lateral_, for organic ions should be lower for ions than for their neutral analogues that were modeled here (unfortunately we do not know any model or experimental data describing *D*_lateral_ for organic ions so this point remains rather speculative). At the same time, *K*_lipw_ for organic ions is generally lower or, in the case of strong uncouplers, at most equally high as for the corresponding neutral chemicals [63]. Thus, we conclude that the contribution of the ionic species to the overall lateral permeability can most likely be ignored and that there is actually no indication from our work for the lateral pathway to be relevant for the description of *P*_app_, at all. For future modeling of *P*_app_ we would exclude the lateral pathway because due to its rather uncertain input parameters it is not likely to improve the predictions.

## Conclusion

The model suggested here can successfully predict experimental *P*_app_ values measured with Caco-2 or MDCK monolayers. With an RMSE of 0.5 log units this model has an uncertainty that is not substantially higher than that from experimental data for a given chemical measured by different researchers. According to our model predictions, roughly 50% of the investigated data were dominated by the paracellular route while the rest permeated through the cytosolic route. On the cytosolic route, by far the most chemicals found their main resistance in the aqueous layers, i.e., unstirred water layers on apical and basolateral side, cytosol and water filled filter pores. This stands somewhat in contrast to the literature, where authors often try to relate measured *P*_app_ values to the intrinsic membrane permeability, *P*_m_, of a chemical, (see, e.g., the major efforts to excerpt *P*_m_ from *P*_app_ (Caco-2/MDCK) [9]). The relative importance of the various resistances must be considered when one tries to transfer experimental *P*_app_ data or our model predictions to scenarios other than in-vitro Caco-2/MDCK tests. In the BBB, e.g., paracellular permeability is usually neglected due to the tightness of the tight junctions between the endothelial cells. Also, the BBB has a much smaller *UWL* compared to a mildly or non-stirred Caco-2/MDCK assay, but it comprises additional resistances due to astrocytic endfeets and a thick basement membrane [64]. Thus one cannot expect to find a direct correlation between experimental *P*_app_ values and the penetration through the BBB for the same chemicals. Experimental *P*_app_ data should be more relevant for judging intestinal absorption efficiency and, of course, our model could be applied in this question as well. The relative importance of the paracellular pathway and the influence of the *ABL* in the intestines might in fact be mimicked quite well by Caco-2 cell tests. However, one has to keep in mind that the fraction absorbed does not only depend on passive permeability but also on active transport, solubility and the presystemic metabolism. All these processes are not represented by experimental *P*_app_ values or our model.

## Supporting information

S1 Supporting Information Supporting information. List of abbreviations used in the manuscript, Thermodynamic coefficients used for the modelling, Excluded *P*_app_ data, Paracellular permeability, *P*_para_, Permeability through the membrane *P*_m_−BLM dataset, Correlation between *P*_m_ and *K*_lipw_ for the BLM dataset, Correlation between *P*_app_ (calc) and *P*_app_ (exp) using only experimentally derived Abraham descriptors, Model parameters.
(DOCX)
