# Supplementary material for: Predicting apparent passive permeability of Caco-2 and MDCK cell-monolayers: A mechanistic model
Source: PLoS One. 2017 Dec 27;12(12):e0190319. doi: 10.1371/journal.pone.0190319 (PMC5744993; doi:10.1371/journal.pone.0190319)
Supplement: S1 Supporting Information — List of abbreviations used in the manuscript, Thermodynamic coefficients used for the modelling, Excluded Papp data, Paracellular permeability, Ppara, Permeability through the membrane Pm−BLM dataset, Correlation between Pm and Klipw for the BLM dataset, Correlation between Papp (calc) and Papp (exp) using only experimentally derived Abraham descriptors, Model parameters. (DOCX) [file pone.0190319.s001.docx]

# Supporting Information

# Predicting apparent passive permeability of Caco-2 and MDCK cell-monolayers: a mechanistic model

## List of abbreviations used in the manuscript

| **abbreviation** | **meaning** |
| --- | --- |
| *ABL* | aqueous boundary layers, consist of several serial and parallel resistances (Fig 1) |
| BBB | blood-brain barrier |
| BLM | black lipid membrane |
| Caco‑2 | human intestinal epithelial cells, originated from human colorectal carcinoma cells |
| c_x_ | concentration of all species in compartment x (i.e., donor or acceptor) |
| *D*_aq_ | aqueous diffusion coefficient |
| *D*_bar_ | diffusion coefficient in the barrier |
| *D*_hex_ | diffusion coefficient in hexadecane |
| *D*_lateral_ | lateral membrane diffusion coefficient |
| *E* | electric field in paracellular pores |
| $F\left( \frac{r_{hydr}}{r_{pore}} \right)$ | Renkin hydrodynamic sieving -function for cylindrical water channels |
| *f*_neutral_ | fraction of the chemicals that exists as neutral species at the experimental pH of 7.4 |
| *f*_x_ | fraction of the chemicals that exists as species x (being neutral, anionic, cationic, dianionic, dicationic or zwitterionic) at the experimental pH of 7.4 |
| *h*_bar_ | thickness of the barrier |
| *h*_cytosol_ | thickness of the cytosol |
| *h*_filter_ | thickness of the polycarbonate filter that serves as the supporting material upon which the cell lines are grown |
| *h*_hex.-like_ | thickness of the hexadecane-like interior of the membrane (approx. 15 Å) |
| *h*_lateral_ | length of the lateral diffusion pathway |
| *h*_m_ | height of the membrane |
| *h*_UWL_ | thickness of the UWL |
| *k*_B_ | Boltzmann constant |
| *K*_barw_ | equilibrium partition coefficient between the barrier and water |
| *K*_hexw_ | equilibrium hexadecane water partition coefficient |
| *K*_lipw_ | equilibrium liposome-water partition coefficient (which is taken as a surrogate for the biological membrane-water partition coefficient) |
| MDCK | Madin−Darby Canine Kidney cells |
| *MW* | molecular weight |
| *P*_cytosol_ | permeability through the Cytosol |
| $P_{cytosol}^{total}$ | cytosolic permeation pathway, comprised of P_UWL_(apical), P_m_(apical), P_cytosol_, P_m_(basal), P_filter_, and P_UWL_(basal) |
| $P_{lateral}^{total}$ | lateral permeation pathway, comprised of P_UWL_(apical), P_lateral_, P_filter_, and P_UWL_(basal) |
| *P*_para_ | paracellular permeability (pH dependent) |
| $P_{para}^{total}$ | paracellular permeation pathway, comprised of P_UWL_(apical), P_para_, P_filter_ and P_UWL_(basal) |
| *P_UWL_* | permeability of the UWL as a whole (i.e., apical and basal UWL together) |
| PAMPA | parallel artificial membrane permeability assay |
| *P*_app_ (Caco-2, pH 7.4) | apparent permeability through Caco-2 cell monolayer at pH 7.4 |
| *P*_app_ (Caco-2/MDCK) | apparent permeability through cell monolayers (from Caco-2 and MDCK cells in this work) - for the sake of brevity also shortly Papp |
| *P*_app_ | short form for *P*_app_ (Caco-2/MDCK) |
| *P*_app_ (MDCK, pH 7.4) | apparent permeability through MDCK cell monolayer at pH 7.4 |
| *P*_bar_ | permeability of barrier bar |
| *P*_lateral_ | lateral permeability along the membrane |
| *R*_cytosol_ | resistance of cytosol |
| $R_{cytosol}^{total}$ | resistance for the cytosolic permeation pathway |
| R_filter | resistance of the polycarbonate filter that serves as the supporting material upon which the cell lines are grown |
| *R*_lateral_ (pH 7.4) | pH dependent lateral resistance |
| $R_{lateral}^{total}$ | resistance for the lateral permeation pathway |
| *R_m_* (apical, pH 7.4) | resistance of apical cell membrane at pH 7.4 |
| *R_m_* (basal, pH 7.4) | resistance of basolateral cell membrane at pH 7.4 |
| *R*_para_ (pH 7.4) | pH dependent paracellular resistance |
| $R_{para}^{total}$ | resistance for the paracellular permeation pathway |
| *R*_UWL_ | resistance of the UWL as a whole (i.e., apical and basal UWL together) |
| *R*_UWL_ (apical) | resistance of apical UWL |
| *R*_UWL_ (basal) | resistance of basal UWL |
| R² | coefficient of determination |
| *R*_bar_ | resistance of barrier bar |
| *r*_hydr_ | hydrodynamic radius |
| *R*_lateral_ | lateral resistance along the membrane |
| *R*_m_ | membrane resistance |
| RMSE | root-mean square error |
| rpm | rounds per minute |
| *r*_pore_ | paracellular pore radius |
| *S* | surface area of the polycarbonate filter that serves as the supporting material upon which the cell lines are grown |
| *S*_bar_ | area across which diffusional transport occurs |
| SI | supporting information |
| *T* | absolute temperature (here 310 K) |
| *UWL* | stagnant, unstirred water layer |
| $v$ | stirring speed (in rpm) |
| *α* | empirical constant for determination of *R*_UWL_ |
| δ | tortuous path length in paracellular permeability |
| Δϕ | potential drop along paracellular pores |
| *ε*_bar_ | actual diffusive area/*S*_bar_ |
| *ε*_filter_ | filter porosity |
| ε_para_ | porosity of paracellular channels |
| κ | constant of 0.037414mV^‑1^ at 37°C for the determination of *P*_para_ |
| φ | factor accounting for the limitation of space available for lateral diffusion |
| *Χ_UWL_* | constant derived from the aqueous diffusivity of the solute to the power of 2/3, the kinematic viscosity (to the power of -1/6) and geometrical factors of the permeation cell |

## Thermodynamic coefficients used for the modelling

### Partition coefficients

#### Phospholipid liposome-water partitioning, $\boldsymbol{K}_{\boldsymbol{lipw}}$

We used a calibrated predictive model from the literature [1] to describe the liposome-water partition coefficient, *K*_lipw_, which we take as a surrogate for the partitioning to the Caco-2/MDCK cell membrane for the description of the lateral permeability pathway:

$logK_{lipw}=0.74E-0.72S+0.11A-3.63B+3.30V+0.29$ (SI -

Eq. (SI - 1) has been fitted for 37 °C with n=131, SD=0.275, R²=0.978. The model details can be found in a number of reviews, e.g., reference [2]. We used the UFZ-LSER database [3] in order to get a maximum amount of experimentally determined solute descriptors, E (excess molar refraction), S (dipolarity/polarizability parameter), A (solute H-bond acidity), B (solute H-bond basicity), and V (molar volume). For cases where no experimental solute descriptors were available we used the UFZ-QSPR, available free of charge from the same source.

#### Hexadecane-water partitioning, $\boldsymbol{K}_{\boldsymbol{hexw}}$

A similar model is used to describe the hexadecane-water partition coefficient, *K*_hexw_, at 25°C [4].

$logK_{hexw}=0.67E-1.62S-3.59A-4.87B+4.43V+0.09$ (SI -

*K*_hexw_ is used to describe the partitioning into the membrane interior in order to model the intrinsic membrane permeability, *P*_m_. In the case of the BLM dataset we also used, if available, experimental *K*_hexw_ values, in order to reduce uncertainty in the model input parameters as much as possible.

### Diffusion coefficients

#### Diffusion coefficients in water, $\boldsymbol{D}_{\boldsymbol{aq}}$

The diffusion coefficients in water at 25°C, *D*_aq_(25°C), were predicted based on the respective molecular weight, $MW$, of the chemical with the following formula from the literature (R²=0.94, n=147) [5]:

$D_{aq}(25^{\circ}C) ={10}^{-4.13-0.453*logMW}$ (SI -

In order to account for the experimental temperature of 37 °C in the permeability tests, *D*_aq_(25°C) values were multiplied with a temperature correction factor of 1.348 [6].

#### Diffusion coefficients in hexadecane, $\boldsymbol{D}_{\boldsymbol{hex}}$

We assumed the diffusion coefficients in hexadecane (which we take as a surrogate for the membrane interior), *D*_hex_, to be one tenth of *D*_aq_(37°C), based on experimental findings [7] and molecular dynamics simulations [8].

#### Lateral membrane diffusion, $\boldsymbol{D}_{\boldsymbol{lateral}}$

The lateral diffusion coefficient, *D*_lateral_, occurs at significantly different rates compared to transbilayer diffusion (which we described using *D*_hex_), because of different underlying mechanisms [9]. We used the formula derived by Johnson et al. [10] to calculate *D*_lateral_

$D_{lateral}(27^{\circ}C) =a*{MW}^{-b}+\left( \frac{k_{b}T}{4\piƞ_{0}h_{m}} \right)\left[ ln\left( \frac{ƞ_{0}h_{m}}{ƞr_{c}} \right)-\gamma_{e} \right]$ (SI -

Where *a* and *b*, which qualify the influence of the *MW*, are empirical parameters (1.45 *10^-4^ cm²/s and ‑1.32, respectively); *k*_b_ is the Boltzmann constant, *T* is the temperature (303 K), *ƞ*_0_ is the effective viscosity of the bilayer (*ƞ*_0_ = 0.75 P = 0.75 * 0.1 Pa·s) and *ƞ* is the viscosity of the surrounding aqueous medium (*ƞ* = 0.01 P = 0.01 * 0.1 Pa·s), *h*_mem_ is the thickness of the membrane (55 Å), *r*_c_ is the solute radius and γ_e_ is Euler's constant (0.5772). The solute radius *r*_c_ can be described [10] via

$r_{c}=\sqrt{MW/ \left( \pi h_{m}N_{a}\rho\right)}$ (SI -

where *N*_a_ is the Avogadro’s number and ρ is the density of the solute.

### Calculation of the pH-dependent fractionation

JChem for Excel, version 15.10.2600.341 (Copyright 2008-2015 ChemAxon Ltd. <https://www.chemaxon.com/>) has been used to predict the pH dependent fraction of neutral species, using a smiles code as input. The program is based on the Hammett-Taft approach and makes additional use of calculated atomic charges. In an investigation of 211 discovery (druglike) chemicals, JChem performed equally well as ACD and the topological method MoKa [11].

## Excluded $\boldsymbol{P}_{\boldsymbol{ap}\boldsymbol{p}}$ data

We excluded one chemical, Cyclosporin A (log *P*_app_ (exp) = -5.26 [12,13]) because its solute descriptors cannot reliably be predicted with the LSER-QSAR [3] due to its high molecular mass of 1203.

## Paracellular permeability, $\boldsymbol{P}_{\boldsymbol{para}}$

Paracellular permeability modeled according to [14] has a noteworthy influence only on the *P*_app_ of those chemcicals where the neutral fraction is predicted to be zero, which is not surprising, because [14] use a negative Potential of 59 mV, while [15] use a negative Potential of 30 mV.

## Permeability through the membrane $\boldsymbol{P}_{\boldsymbol{m}}$ – BLM dataset

In the BLM experiments collected from the literature, single artificial bilayer membranes were formed with the brush technique [16] with a decane solution of egg phosphatidylcholine (typically 20 mg/mL) or equivalent phospholipids. The bilayers span over a ~1-2mm² hole in a Teflon sheet, separating two magnetically stirred Teflon chambers (each typically 3 mL big). After the membrane turns optically black, the membrane electrical resistance and capacitance can be determined from the current measured for a specific applied voltage. In the permeability experiments, the unstirred water layer ($UWL$) must explicitly be taken into account: in the so-called radiotracer method, symmetrical conditions are used to measure one-way tracer fluxes ($J$) under constant sink conditions. The concentration ratios of the uncharged $\left[ AH \right]$ over the charged form $\left[ A^{-} \right]$ (in the case of acids) are varied over several orders of magnitude with different pH values. Assuming that only the uncharged species permeates the membrane to a significant degree, and assuming that the UWL permeability ($P_{UWL}$) is the same for ionic and neutral species, then both membrane permeability ($P_{m}$) and $P_{UWL}$ can be calculated from a plot of the measured fluxes at different pH values (i.e., at different speciation) [17]

$\frac{1}{J}=\frac{1}{P_{UWL}(\left[ A \right]+\left[ A^{-} \right])}+\frac{1}{P_{m}(\left[ A \right])}$ (SI -

Eq. 13 of the main article implies a highly buffered solution so that the pH in the $UWL$ can be treated as constant [18]. A different and more sophisticated method, the pH electrode technique, accounts for the measured pH gradients on both sides of the membrane that result from proton flux between the bulk phases and the membrane that is induced by the dissociation reactions at the membrane surface where only the neutral species is considered being able to permeate [19]. While the pH electrode technique was initially solely used to describe the pH gradients generated by the weak acids and bases themselves in the unbuffered solutions [19], the buffer was later taken into account for the limiting case of low membrane fluxes [20]. This latter method was markedly expanded by including also the different buffer concentrations and describing the pH profiles on both sides of the membrane theoretically as well as experimentally (with micro-pH electrodes) [21].

Table 1. Compilation of experimental membrane permeability data derived from BLM experiments:

| **Compound** | **log P_m_ (BLM) exp** | **reference** | **MW** | **log P_m_ (BLM) calc** |
| --- | --- | --- | --- | --- |
| 1,2-ethanediol | -4.06 | [22] | 62.07 | -3.89 |
| 1,2-propanediol | -3.55 | [23] | 76.10 | -3.35 |
| 1,4-butanediol | -3.58 | [22,23] | 90.12 | -3.56 |
| 2',3'-Dideoxyadenosine | -4.20 | [24] | 235.25 | -3.35 |
| 2'-Deoxyadenosine | -6.03 | [24] | 251.25 | -6.04 |
| 9-Anthroic acid | 0.51 | [24] | 222.24 | 2.03 |
| acetamide | -3.68 | [22,23,25] | 59.07 | -3.79 |
| acetic acid | -2.21 | [21,24,26] | 60.05 | -2.54 |
| Adenine | -4.86 | [24] | 135.13 | -3.77 |
| alpha-carbamoyl-p-toluic acid | -4.39 | [27] | 179.18 | -5.15 |
| alpha-carboxy-p-toluic acid | -3.74 | [27] | 180.16 | -4.36 |
| alpha-chloro-p-toluic acid | -0.19 | [27] | 170.59 | -0.20 |
| alpha-cyano-p-toluic acid | -1.57 | [27] | 161.16 | -1.93 |
| alpha-hydroxy-p-toluic acid | -2.80 | [25] | 152.15 | -3.57 |
| alpha-methoxy-p-toluic acid | -0.46 | [27] | 166.18 | -0.79 |
| alpha-Naphthoic acid | 0.36 | [24] | 172.18 | 1.06 |
| benzoic acid | -0.25 | [24,26] | 122.12 | -0.53 |
| beta-Naphthoic acid | 1.23 | [24] | 172.18 | 0.76 |
| butyric acid | -1.05 | [19,23,24,26] | 88.11 | -1.25 |
| carbonylcyanide m-chlorophenylhydrazone | 1.04 | [28] | 204.62 | 0.11 |
| codeine | -0.85 | [23] | 299.37 | -0.81 |
| glycerol | -5.27 | [23] | 92.09 | -4.90 |
| hexanoic acid | 0.04 | [26] | 116.16 | -0.10 |
| histamine | -4.46 | [29] | 110.14 | -3.75 |
| hydrocortisone | -3.25 | [24] | 362.47 | -3.08 |
| hydrocortisone-21-pimelamide | -3.74 | [24] | 502.65 | -5.37 |
| nitric acid | -3.04 | [30] | 63.01 | -3.28 |
| phloretin | -3.62 | [31] | 274.27 | -3.70 |
| Prednisolone | -3.82 | [24] | 360.45 | -2.61 |
| propionic acid | -1.59 | [26] | 74.08 | -1.79 |
| p-toluic acid | 0.04 | [25] | 136.15 | 0.14 |
| Salicylic acid | -0.05 | [18,26,32] | 138.12 | -0.49 |
| triethylamine | 0.03 | [33] | 101.19 | 1.52 |
| theophylline | -3.54 | [29] | 180.17 | -3.88 |
| thiocyanic acid | 0.41 | [34] | 59.09 | 0.40 |
| tryptamine | -0.74 | [29] | 160.22 | -0.68 |
| urea | -5.40 | [22] | 60.06 | -5.67 |

## Correlation between $\boldsymbol{P}_{\boldsymbol{m}}$ and $\boldsymbol{K}_{\boldsymbol{lipw}}$ for the BLM dataset


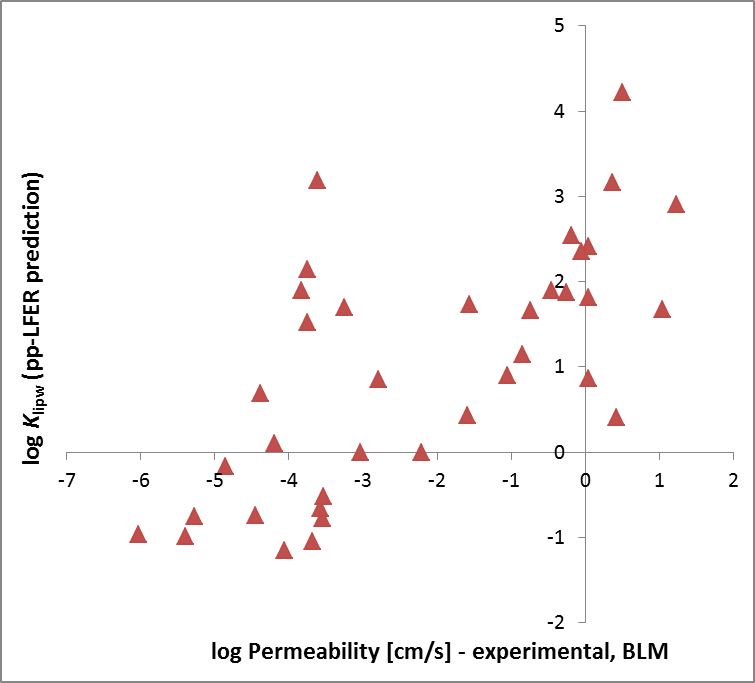


Fig 1. Correlation between *P*_m_ and *K*_lipw_ for the BLM Dataset. The *K*_lipw_ values were calculated with pp-LFER Eq. SI - 1.

## Correlation between $\boldsymbol{P}_{\boldsymbol{app}}\boldsymbol{(calc)}$ and $\boldsymbol{P}_{\boldsymbol{app}}\boldsymbol{(}\boldsymbol{exp}\boldsymbol{)}$ using only experimentally derived Abraham descriptors


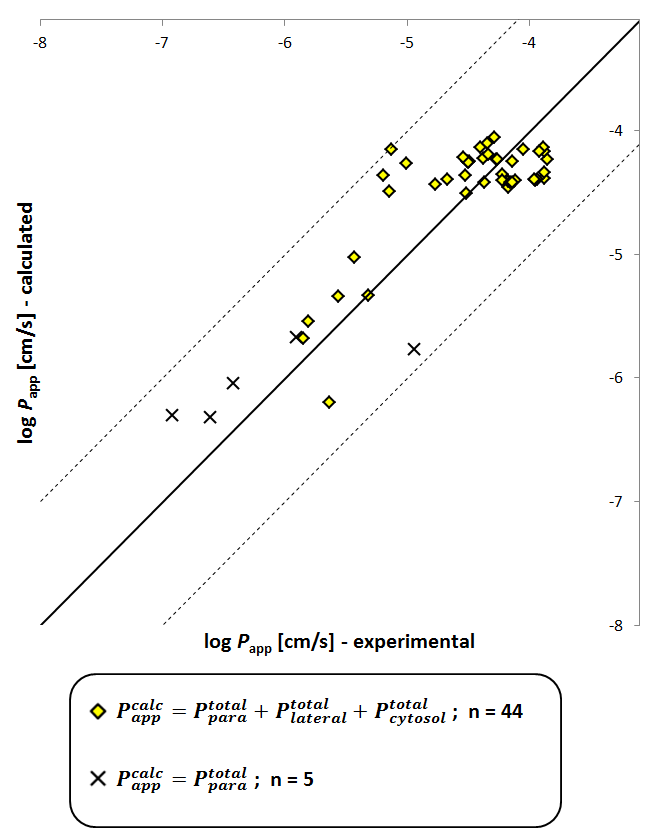


Fig 2. Correlation between *P*_app_(calc) and *P*_app_ (exp) at pH 7.4 (analogous to Fig. 3 of the manuscript) using only experimentally derived Abraham descriptors.

Yellow diamonds were modeled with three optional permeation routes (paracellular, cytosolic and lateral), while x-shaped black crosses are chemicals that were a priori limited to the paracellular route, because they were estimated to be 100% ionized. The model has an overall predictive quality of RMSE=0.38, R²=0.75.

## Model parameters

Table 2. Fractionation at pH 7.4 according to JChem

| **name** | **fraction neutral** | **fraction anion** | **fraction cation** | **fraction zwitterion** | **fraction dication** | **fraction dianion** |
| --- | --- | --- | --- | --- | --- | --- |
| acebutolol | 0.0068 | 0 | 0.9932 | 0 | 0 | 0 |
| acetaminophen | 0.9915 | 0.0085 | 0 | 0 | 0 | 0 |
| acetylsalicylic acid | 0.0001 | 0.9999 | 0 | 0 | 0 | 0 |
| alprenolol | 0.0054 | 0 | 0.9946 | 0 | 0 | 0 |
| Amantadine | 0.0005 | 0 | 0.9995 | 0 | 0 | 0 |
| Amiloride | 1 | 0 | 0 | 0 | 0 | 0 |
| Amitriptyline | 0.0043 | 0 | 0.9957 | 0 | 0 | 0 |
| Amodiaquine | 0.0055 | 0 | 0.88 | 0.0121 | 0.1017 | 0 |
| Antipyrine | 1 | 0 | 0 | 0 | 0 | 0 |
| Antipyrine | 1 | 0 | 0 | 0 | 0 | 0 |
| Caffeine | 1 | 0 | 0 | 0 | 0 | 0 |
| Caffeine | 1 | 0 | 0 | 0 | 0 | 0 |
| Chloramphenicol | 0.9513 | 0.0487 | 0 | 0 | 0 | 0 |
| Chloroquine | 0.0007 | 0 | 0.5648 | 0 | 0.434 | 0 |
| Chloroquine | 0.0007 | 0 | 0.5648 | 0 | 0.434 | 0 |
| Clemastine | 0.007 | 0 | 0.993 | 0 | 0 | 0 |
| Clonidine | 0.1469 | 0 | 0.8531 | 0 | 0 | 0 |
| Clozapine | 0.5281 | 0 | 0.4716 | 0 | 0.0002 | 0 |
| corticosterone | 1 | 0 | 0 | 0 | 0 | 0 |
| Desipramine | 0.0024 | 0 | 0.9976 | 0 | 0 | 0 |
| dexamethasone | 1 | 0 | 0 | 0 | 0 | 0 |
| Doxepin | 0.0043 | 0 | 0.9957 | 0 | 0 | 0 |
| Ethosuximide | 0.9995 | 0.0005 | 0 | 0 | 0 | 0 |
| Fluoxetine | 0.0039 | 0 | 0.9961 | 0 | 0 | 0 |
| fluparoxan | 0.0078 | 0 | 0.9922 | 0 | 0 | 0 |
| Fluvastatin | 0.0014 | 0.9986 | 0 | 0 | 0 | 0 |
| Genistein | 0.1038 | 0.7409 | 0 | 0 | 0 | 0.1555 |
| Gly-Pro | 0.9999 | 0.0001 | 0 | 0 | 0 | 0 |
| hydrocortisone | 1 | 0 | 0 | 0 | 0 | 0 |
| Hydroxyzine | 0.4732 | 0 | 0.5268 | 0 | 0 | 0 |
| Imipramine | 0.0157 | 0 | 0.9843 | 0 | 0 | 0 |
| Isocarboxazid | 0.999 | 0.001 | 0 | 0 | 0 | 0 |
| ketoprofen | 0.0003 | 0.9997 | 0 | 0 | 0 | 0 |
| labetalol | 0.0041 | 0.0007 | 0.8163 | 0.1788 | 0 | 0 |
| lamotrigine | 0.9711 | 0 | 0.0289 | 0 | 0 | 0 |
| Lansoprazole | 0.9882 | 0.0112 | 0.0006 | 0 | 0 | 0 |
| Levofloxacin | 0.0141 | 0.9398 | 0.0007 | 0.0454 | 0 | 0 |
| methylprednisolone | 1 | 0 | 0 | 0 | 0 | 0 |
| metoprolol | 0.0054 | 0 | 0.9946 | 0 | 0 | 0 |
| minoxidil | 0.9999 | 0 | 0.0001 | 0 | 0 | 0 |
| Mirtazapine | 0.8423 | 0 | 0.1562 | 0 | 0.0014 | 0 |
| Morphine | 0.0155 | 0 | 0.9813 | 0.0031 | 0 | 0 |
| naproxen | 0.0006 | 0.9994 | 0 | 0 | 0 | 0 |
| Nicotine | 0.0624 | 0 | 0.9376 | 0 | 0 | 0 |
| Ofloxacin | 0.0141 | 0.9398 | 0.0007 | 0.0454 | 0 | 0 |
| Omeprazole | 0.9851 | 0.0126 | 0.0023 | 0 | 0 | 0 |
| ondansetron | 0.532 | 0 | 0.468 | 0 | 0 | 0 |
| oxyprenolol | 0.9998 | 0.0002 | 0 | 0 | 0 | 0 |
| Pantoprazole | 0.9825 | 0.0174 | 0.0001 | 0 | 0 | 0 |
| Pemoline | 1 | 0 | 0 | 0 | 0 | 0 |
| Pergolide | 0.0081 | 0 | 0.9919 | 0 | 0 | 0 |
| Phenelzine | 0.986 | 0 | 0.014 | 0 | 0 | 0 |
| phenytoin | 0.9242 | 0.0758 | 0 | 0 | 0 | 0 |
| Phenytoin | 0.9242 | 0.0758 | 0 | 0 | 0 | 0 |
| pindolol | 0.0054 | 0 | 0.9946 | 0 | 0 | 0 |
| practolol | 0.0054 | 0 | 0.9946 | 0 | 0 | 0 |
| Praziquantel | 1 | 0 | 0 | 0 | 0 | 0 |
| Prazosine | 0.5894 | 0 | 0.4106 | 0 | 0 | 0 |
| propranolol | 0.0054 | 0 | 0.9946 | 0 | 0 | 0 |
| propylthiouracil | 0.4686 | 0.5242 | 0 | 0 | 0 | 0 |
| Pumafentrine | 0.1898 | 0.8102 | 0 | 0 | 0 | 0 |
| Quinidine | 0.0221 | 0 | 0.9775 | 0 | 0.0004 | 0 |
| Quinine | 0.0221 | 0 | 0.9775 | 0 | 0.0004 | 0 |
| Ranitidine | 0.0286 | 0.0012 | 0.5406 | 0.1459 | 0.2836 | 0 |
| Selegiline | 0.0506 | 0 | 0.9494 | 0 | 0 | 0 |
| sotalol | 0.0059 | 0 | 0.9907 | 0.0034 | 0 | 0 |
| sumatriptan | 0.0071 | 0 | 0.9928 | 0.0001 | 0 | 0 |
| Tacrine | 0.0272 | 0 | 0.9728 | 0 | 0 | 0 |
| Temazepam | 0.9995 | 0.0005 | 0 | 0 | 0 | 0 |
| theophylline | 0.7223 | 0.2777 | 0 | 0 | 0 | 0 |
| Thiabendazole | 0.9982 | 0.0013 | 0.0005 | 0 | 0 | 0 |
| timolol | 0.0043 | 0 | 0.9956 | 0 | 0 | 0 |
| Tolafentrine | 0.139 | 0.0415 | 0.6344 | 0.1844 | 0.0002 | 0 |
| Trazodone | 0.6722 | 0 | 0.3278 | 0 | 0 | 0 |
| trimethoprim | 0.6367 | 0.3633 | 0 | 0 | 0 | 0 |
| Urea | 1 | 0 | 0 | 0 | 0 | 0 |
| verapamil | 0.0052 | 0 | 0.9948 | 0 | 0 | 0 |
| Verapamil | 0.0052 | 0 | 0.9948 | 0 | 0 | 0 |
| warfarin | 0.0142 | 0.9858 | 0 | 0 | 0 | 0 |
| Warfarin | 0.0142 | 0.9858 | 0 | 0 | 0 | 0 |
| Zidovudine | 0.9964 | 0.0029 | 0.0007 | 0 | 0 | 0 |
| acyclovir | 0.7939 | 0.2058 | 0 | 0 | 0 | 0 |
| Atenolol | 0.0054 | 0 | 0.9946 | 0 | 0 | 0 |
| Cefotaxime | 0.0001 | 0.9992 | 0 | 0 | 0 | 0 |
| Cefuroxime | 0.0001 | 0.9997 | 0 | 0 | 0 | 0 |
| Cephalothin | 0.0002 | 0.9998 | 0 | 0 | 0 | 0 |
| Cephapirin | 0.0001 | 0.996 | 0 | 0.0038 | 0 | 0 |
| Chlorothiazide | 0.9805 | 0.0194 | 0 | 0 | 0 | 0 |
| Ciprofloxacin | 0.0011 | 0.049 | 0.0211 | 0.9288 | 0 | 0 |
| Furosemide | 0.0007 | 0.9956 | 0 | 0 | 0 | 0 |
| Glycerol | 1 | 0 | 0 | 0 | 0 | 0 |
| hydrochlorothiazide | 0.9798 | 0.0202 | 0 | 0 | 0 | 0 |
| Lactic acid | 0.0002 | 0.9998 | 0 | 0 | 0 | 0 |
| Mannitol | 1 | 0 | 0 | 0 | 0 | 0 |
| nadolol | 0.0043 | 0 | 0.9956 | 0 | 0 | 0 |
| Norfloxacin | 0.0012 | 0.049 | 0.0217 | 0.9281 | 0 | 0 |
| penicillin v | 0.0001 | 0.9999 | 0 | 0 | 0 | 0 |
| sulpiride | 0.0914 | 0.0001 | 0.9072 | 0.0013 | 0 | 0 |
| Saquinavir (+ Ritonavir inhibitor) | 0.0777 | 0 | 0.9222 | 0 | 0 | 0 |
| Quercetin | 0.0647 | 0.6793 | 0 | 0 | 0 | 0.256 |
| Cefamandole | 0.0001 | 0.9998 | 0 | 0 | 0 | 0 |
| Cefoperazone | 0.0001 | 0.9914 | 0 | 0 | 0 | 0 |
| Cefoxitin | 0.0002 | 0.9996 | 0 | 0 | 0 | 0 |
| Cimetidine | 0.8807 | 0 | 0.1191 | 0 | 0.0002 | 0 |
| Cimetidine | 0.8807 | 0 | 0.1191 | 0 | 0.0002 | 0 |
| Doxorubicin | 0.0111 | 0.0034 | 0.7951 | 0.1904 | 0 | 0 |
| Erythromycin | 0.0945 | 0 | 0.9054 | 0 | 0 | 0 |
| Mitoxantrone | 0.001 | 0.0001 | 0.9937 | 0.0052 | 0 | 0 |
| netivudine | 0.9909 | 0.0091 | 0 | 0 | 0 | 0 |
| Sucrose | 1 | 0 | 0 | 0 | 0 | 0 |
| terbutaline | 0.0059 | 0.0001 | 0.9662 | 0.0277 | 0 | 0 |
| bupropion | 0.1326 | 0 | 0.8674 | 0 | 0 | 0 |
| Carbamazepine | 1 | 0 | 0 | 0 | 0 | 0 |
| Chlorpromazine | 0.0157 | 0 | 0.9843 | 0 | 0 | 0 |
| Diazepam | 1 | 0 | 0 | 0 | 0 | 0 |
| Diazepam | 1 | 0 | 0 | 0 | 0 | 0 |
| Flavone | 1 | 0 | 0 | 0 | 0 | 0 |
| guanabenz | 0.1418 | 0 | 0.8582 | 0 | 0 | 0 |
| Haloperidol | 0.1845 | 0 | 0.8155 | 0 | 0 | 0 |
| Loxapine | 0.6266 | 0 | 0.3734 | 0 | 0 | 0 |
| Midazolam | 0.8707 | 0 | 0.1292 | 0 | 0 | 0 |
| progesterone | 1 | 0 | 0 | 0 | 0 | 0 |
| Quetiapine | 0.684 | 0 | 0.316 | 0 | 0 | 0 |
| Testosterone | 1 | 0 | 0 | 0 | 0 | 0 |
| Ziprasidone | 0.6723 | 0 | 0.3277 | 0 | 0 | 0 |
| Terfenadine | 0.0233 | 0 | 0.9767 | 0 | 0 | 0 |
| Ritonavir | 1 | 0 | 0 | 0 | 0 | 0 |
| acrivastine | 0 | 0.0556 | 0.0002 | 0.9442 | 0 | 0 |
| Alanine | 0 | 0.0083 | 0 | 0.9917 | 0 | 0 |
| amoxicillin | 0 | 0.5944 | 0 | 0.3967 | 0 | 0 |
| AZT | 0 | 0.0007 | 0 | 0 | 0 | 0.9992 |
| bretylium | 0 | 0 | 1 | 0 | 0 | 0 |
| Cefaclor | 0 | 0.5974 | 0 | 0.4025 | 0 | 0 |
| Cefadroxil | 0 | 0.5944 | 0 | 0.3967 | 0 | 0 |
| cefatrizine | 0 | 0.5954 | 0 | 0.3913 | 0 | 0.0131 |
| Cefazolin | 0 | 0.9996 | 0 | 0 | 0 | 0 |
| Cefsulodine | 0 | 0.9998 | 0 | 0 | 0 | 0 |
| Ceftazidime | 0 | 0.9989 | 0 | 0.0006 | 0 | 0 |
| Ceftriaxon | 0 | 0.012 | 0 | 0 | 0 | 0.9879 |
| Cephalexin | 0 | 0.5973 | 0 | 0.4025 | 0 | 0 |
| Cephaloglycin | 0 | 0.5974 | 0 | 0.4025 | 0 | 0 |
| Cephaloridine | 0 | 0.0001 | 0.0001 | 0.9998 | 0 | 0 |
| Cephradine | 0 | 0.3893 | 0.0001 | 0.6105 | 0 | 0 |
| Cetirizine | 0 | 0.4884 | 0.0001 | 0.5114 | 0 | 0 |
| gabapentin | 0 | 0.003 | 0.0017 | 0.9953 | 0 | 0 |
| Gatifloxacin | 0 | 0.044 | 0.0181 | 0.9371 | 0 | 0 |
| L-Dopa | 0 | 0.0216 | 0 | 0.9782 | 0 | 0.0002 |
| Leucine | 0 | 0.0075 | 0 | 0.9924 | 0 | 0 |
| lisinopril | 0 | 0.1976 | 0.0002 | 0.8017 | 0 | 0.0003 |
| Lomefloxacin | 0 | 0.0473 | 0.0164 | 0.9355 | 0 | 0 |
| loracarbef-D | 0 | 0.5974 | 0 | 0.4025 | 0 | 0 |
| Methotrexate | 0 | 0.0004 | 0 | 0 | 0 | 0.9996 |
| Moxifloxacin | 0 | 0.0092 | 0.0188 | 0.9718 | 0 | 0 |
| olsalazine | 0 | 0.0001 | 0 | 0 | 0 | 0.9999 |
| Phenylalanine | 0 | 0.0089 | 0 | 0.9911 | 0 | 0 |
| Salicylic acid | 0 | 1 | 0 | 0 | 0 | 0 |
| Sulfasalazine | 0 | 0.1812 | 0 | 0 | 0 | 0.8187 |
| Taurocholic acid | 0 | 1 | 0 | 0 | 0 | 0 |

Table 3. Calculated diffusion coefficients and experimental apparent permeabilities from the following literature [12,13,35–43].

| **name** | **Diff_lateral_ [cm²/s] 30°C** | **Diff_aq_ [cm²/s] (37°C)** | ***P*_app_ (MDCK) [cm/s]** | ***P*_app_ (Caco-2) [cm/s]** | **log *P*_app_ MEAN (EXP)** | **references** |
| --- | --- | --- | --- | --- | --- | --- |
| acebutolol | 1.25E-07 | 7.15E-06 | 1.70E-06 | 3.80E-06 | -5.56 | Irvine 1999 |
| acetaminophen | 2.54E-07 | 1.03E-05 | 3.50E-05 | 1.00E-04 | -4.17 | Irvine 1999 |
| acetylsalicylic acid | 2.13E-07 | 9.48E-06 | 7.40E-06 | 2.20E-06 | -5.32 | Irvine 1999 |
| alprenolol | 1.58E-07 | 8.18E-06 | 1.60E-04 | 1.70E-04 | -3.88 | Irvine 1999;Richter 2009 |
| Amantadine | 2.53E-07 | 1.03E-05 | 7.10E-06 |  | -5.15 | Summerfield 2007 |
| Amiloride | 1.71E-07 | 8.49E-06 | 2.54E-06 | 4.26E-06 | -5.47 | Richter 2009 |
| Amitriptyline | 1.45E-07 | 7.80E-06 | 9.80E-06 |  | -5.01 | Summerfield 2007 |
| Amodiaquine | 1.20E-07 | 6.97E-06 |  | 8.05E-05 | -4.09 | Hayeshi 2006 |
| Antipyrine | 2.04E-07 | 9.30E-06 | 1.85E-05 |  | -3.94 | Wang 2005;Irvine 1999;Richter 2009 |
| Antipyrine | 2.04E-07 | 9.30E-06 | 5.66E-05 | 5.12E-05 | -4.27 | Garberg 2005 |
| Caffeine | 1.99E-07 | 9.16E-06 | 2.12E-05 |  | -4.67 | Wang 2005 |
| Caffeine | 1.99E-07 | 9.16E-06 | 5.68E-05 | 5.31E-05 | -4.26 | Garberg 2005 |
| Chloramphenicol | 1.29E-07 | 7.28E-06 | 1.75E-05 | 4.90E-05 | -4.48 | Richter 2009 |
| Chloroquine | 1.29E-07 | 7.31E-06 | 2.40E-05 | 8.87E-06 | -4.78 | Richter 2009 |
| Chloroquine | 1.29E-07 | 7.31E-06 |  | 7.90E-05 | -4.10 | Hayeshi 2006 |
| Clemastine | 1.23E-07 | 7.07E-06 | 4.07E-05 |  | -4.39 | Obradovic 2007 |
| Clonidine | 1.71E-07 | 8.49E-06 | 1.47E-05 |  | -4.83 | Wang 2005 |
| Clozapine | 1.28E-07 | 7.24E-06 | 2.83E-05 |  | -4.55 | Summerfield 2007 |
| corticosterone | 1.22E-07 | 7.05E-06 | 1.40E-04 | 1.20E-04 | -3.89 | Irvine 1999 |
| Desipramine | 1.50E-07 | 7.94E-06 | 3.83E-05 | 8.16E-05 | -4.22 | Richter 2009 |
| dexamethasone | 1.12E-07 | 6.66E-06 | 2.00E-05 | 4.00E-05 | -4.52 | Irvine 1999 |
| Doxepin | 1.44E-07 | 7.77E-06 | 1.63E-05 |  | -4.79 | Summerfield 2007 |
| Ethosuximide | 2.72E-07 | 1.06E-05 | 9.70E-06 |  | -5.01 | Summerfield 2007 |
| Fluoxetine | 1.33E-07 | 7.42E-06 | 6.40E-06 |  | -5.19 | Summerfield 2007 |
| fluparoxan | 1.98E-07 | 9.14E-06 | 2.50E-04 | 2.00E-04 | -3.65 | Irvine 1999 |
| Fluvastatin | 1.08E-07 | 6.52E-06 | 3.07E-05 | 2.69E-05 | -4.54 | Richter 2009 |
| Genistein | 1.49E-07 | 7.89E-06 |  | 1.36E-04 | -3.87 | Hayeshi 2006 |
| Gly-Pro | 2.49E-07 | 1.02E-05 |  | 4.23E-06 | -5.37 | Alsenz 2003 |
| hydrocortisone | 1.18E-07 | 6.91E-06 | 3.10E-05 | 5.60E-05 | -4.36 | Irvine 1999 |
| Hydroxyzine | 1.15E-07 | 6.80E-06 | 3.11E-05 |  | -4.51 | Obradovic 2007 |
| Imipramine | 1.43E-07 | 7.76E-06 | 1.54E-04 | 1.10E-04 | -3.88 | Richter 2009 |
| Isocarboxazid | 1.69E-07 | 8.47E-06 | 1.77E-05 |  | -4.75 | Summerfield 2007 |
| ketoprofen | 1.56E-07 | 8.11E-06 | 1.56E-05 |  | -4.37 | Thiel-Demby 2008;Irvine 1999 |
| labetalol | 1.27E-07 | 7.22E-06 | 2.50E-05 | 7.60E-05 | -4.30 | Irvine 1999 |
| lamotrigine | 1.56E-07 | 8.09E-06 | 8.80E-05 | 1.10E-04 | -4.14 | Irvine 1999;Summerfield 2007 |
| Lansoprazole | 1.17E-07 | 6.85E-06 | 6.90E-05 | 1.50E-04 | -3.96 | Richter 2009 |
| Levofloxacin | 1.19E-07 | 6.92E-06 | 1.64E-06 |  | -5.79 | Robertson 2005 |
| methylprednisolone | 1.16E-07 | 6.81E-06 | 1.60E-05 | 2.50E-05 | -4.69 | Irvine 1999 |
| metoprolol | 1.49E-07 | 7.93E-06 | 4.10E-05 |  | -3.95 | Thiel-Demby 2008;Irvine 1999;Richter 2009 |
| minoxidil | 1.85E-07 | 8.86E-06 | 2.60E-06 |  | -5.59 | Thiel-Demby 2008 |
| Mirtazapine | 1.50E-07 | 7.96E-06 | 3.24E-05 |  | -4.49 | Summerfield 2007 |
| Morphine | 1.42E-07 | 7.70E-06 | 4.05E-06 | 1.04E-05 | -5.14 | Garberg 2005 |
| naproxen | 1.70E-07 | 8.48E-06 | 3.11E-05 |  | -4.17 | Thiel-Demby 2008;Richter 2009 |
| Nicotine | 2.36E-07 | 9.94E-06 | 4.92E-05 | 3.46E-05 | -4.38 | Garberg 2005 |
| Ofloxacin | 1.19E-07 | 6.92E-06 | 1.51E-06 |  | -5.82 | Robertson 2005 |
| Omeprazole | 1.23E-07 | 7.06E-06 | 5.90E-05 | 1.40E-04 | -4.00 | Richter 2009 |
| ondansetron | 1.39E-07 | 7.60E-06 | 1.10E-04 | 1.10E-04 | -3.96 | Irvine 1999 |
| oxyprenolol | 3.29E-07 | 1.15E-05 | 1.30E-04 | 1.60E-04 | -3.84 | Irvine 1999 |
| Pantoprazole | 1.14E-07 | 6.73E-06 | 4.95E-05 | 1.34E-04 | -4.04 | Richter 2009 |
| Pemoline | 2.18E-07 | 9.58E-06 | 4.60E-06 |  | -5.34 | Summerfield 2007 |
| Pergolide | 1.31E-07 | 7.37E-06 | 2.51E-05 |  | -4.60 | Summerfield 2007 |
| Phenelzine | 2.82E-07 | 1.08E-05 | 5.47E-05 |  | -4.26 | Summerfield 2007 |
| phenytoin | 1.57E-07 | 8.14E-06 | 1.20E-04 | 1.60E-04 | -3.85 | Irvine 1999 |
| Phenytoin | 1.57E-07 | 8.14E-06 | 4.61E-05 | 4.45E-05 | -4.34 | Garberg 2005 |
| pindolol | 1.59E-07 | 8.20E-06 | 2.66E-05 |  | -4.22 | Thiel-Demby 2008;Irvine 1999 |
| practolol | 1.50E-07 | 7.94E-06 | 1.30E-06 | 6.10E-06 | -5.43 | Irvine 1999 |
| Praziquantel | 1.32E-07 | 7.39E-06 |  | 1.77E-04 | -3.75 | Hayeshi 2006 |
| Prazosine | 1.14E-07 | 6.73E-06 | 2.56E-05 | 1.54E-05 | -4.69 | Richter 2009 |
| propranolol | 1.53E-07 | 8.04E-06 | 4.47E-05 |  | -3.96 | Thiel-Demby 2008;Irvine 1999;Richter 2009 |
| propylthiouracil | 2.25E-07 | 9.73E-06 | 4.10E-05 | 9.60E-05 | -4.16 | Irvine 1999 |
| Pumafentrine | 9.84E-08 | 6.10E-06 | 4.03E-05 | 7.70E-05 | -4.23 | Richter 2009 |
| Quinidine | 1.28E-07 | 7.26E-06 | 4.32E-05 | 1.10E-04 | -4.12 | Richter 2009 |
| Quinine | 1.28E-07 | 7.26E-06 |  | 8.88E-05 | -4.05 | Hayeshi 2006 |
| Ranitidine | 1.31E-07 | 7.37E-06 | 1.42E-06 | 4.45E-06 | -5.53 | Richter 2009 |
| Selegiline | 2.05E-07 | 9.32E-06 | 4.86E-05 |  | -4.31 | Summerfield 2007 |
| sotalol | 1.47E-07 | 7.86E-06 | 4.70E-06 | 4.20E-06 | -5.35 | Irvine 1999 |
| sumatriptan | 1.38E-07 | 7.58E-06 | 1.90E-06 |  | -5.72 | Irvine 1999 |
| Tacrine | 1.94E-07 | 9.08E-06 | 2.84E-05 |  | -4.55 | Summerfield 2007 |
| Temazepam | 1.36E-07 | 7.52E-06 | 4.21E-05 |  | -4.38 | Summerfield 2007 |
| theophylline | 2.14E-07 | 9.48E-06 | 3.03E-05 |  | -4.52 | Thiel-Demby 2008 |
| Thiabendazole | 1.92E-07 | 9.02E-06 |  | 1.68E-04 | -3.78 | Hayeshi 2006 |
| timolol | 1.31E-07 | 7.35E-06 | 5.50E-05 | 1.00E-04 | -4.11 | Irvine 1999 |
| Tolafentrine | 9.54E-08 | 5.94E-06 | 1.04E-05 | 2.48E-05 | -4.76 | Richter 2009 |
| Trazodone | 1.16E-07 | 6.83E-06 | 3.77E-05 |  | -4.42 | Summerfield 2007 |
| trimethoprim | 1.40E-07 | 7.64E-06 | 5.20E-05 | 8.70E-05 | -4.16 | Irvine 1999 |
| Urea | 7.16E-07 | 1.56E-05 | 2.08E-06 | 6.60E-06 | -5.36 | Garberg 2005 |
| verapamil | 1.01E-07 | 6.23E-06 | 2.63E-05 |  | -4.14 | Thiel-Demby 2008;Richter 2009 |
| Verapamil | 1.01E-07 | 6.23E-06 | 3.14E-05 |  | -4.50 | Garberg 2005 |
| warfarin | 1.33E-07 | 7.43E-06 | 4.40E-05 | 9.60E-05 | -4.15 | Irvine 1999 |
| Warfarin | 1.33E-07 | 7.43E-06 | 3.65E-05 | 4.01E-05 | -4.42 | Garberg 2005 |
| Zidovudine | 1.50E-07 | 7.93E-06 | 6.00E-06 | 2.80E-05 | -4.77 | Irvine 1999 |
| acyclovir | 1.74E-07 | 8.57E-06 | 1.40E-06 |  | -5.84 | Thiel-Demby 2008;Irvine 1999;Richter 2009 |
| Atenolol | 1.50E-07 | 7.94E-06 | 1.90E-07 |  | -5.81 | Wang 2005;Thiel-Demby 2008;Irvine 1999;Richter 2009 |
| Cefotaxime | 1.02E-07 | 6.23E-06 |  | 1.24E-07 | -6.91 | Raeissi 1999 |
| Cefuroxime | 1.07E-07 | 6.43E-06 |  | 1.52E-07 | -6.70 | Raeissi 1999;Wang 2005;Irvine 1999 |
| Cephalothin | 1.12E-07 | 6.63E-06 |  | 3.95E-07 | -6.40 | Raeissi 1999 |
| Cephapirin | 1.07E-07 | 6.44E-06 |  | 3.93E-07 | -6.41 | Raeissi 1999 |
| Chlorothiazide | 1.39E-07 | 7.58E-06 | 2.35E-07 |  | -6.55 | Wang 2005;Irvine 1999 |
| Ciprofloxacin | 1.27E-07 | 7.19E-06 | 4.50E-07 |  | -6.35 | Robertson 2005 |
| Furosemide | 1.27E-07 | 7.20E-06 | 3.15E-08 |  | -6.01 | Wang 2005;Irvine 1999;Richter 2009 |
| Glycerol | 4.33E-07 | 1.28E-05 | 1.73E-06 | 8.60E-06 | -5.29 | Garberg 2005 |
| hydrochlorothiazide | 1.38E-07 | 7.55E-06 | 7.60E-07 |  | -6.05 | Thiel-Demby 2008;Irvine 1999 |
| Lactic acid | 4.45E-07 | 1.30E-05 | 5.75E-06 | 1.10E-06 | -5.47 | Garberg 2005 |
| Mannitol | 2.11E-07 | 9.43E-06 |  | 2.28E-06 | -5.85 | Alsenz 2003;Irvine 1999 |
| nadolol | 1.33E-07 | 7.42E-06 | 8.80E-07 |  | -6.05 | Thiel-Demby 2008;Irvine 1999 |
| Norfloxacin | 1.30E-07 | 7.32E-06 | 3.30E-07 |  | -6.48 | Robertson 2005 |
| penicillin v | 1.22E-07 | 7.01E-06 | 1.50E-07 | 1.70E-07 | -6.80 | Irvine 1999 |
| sulpiride | 1.24E-07 | 7.10E-06 | 8.90E-07 |  | -5.82 | Thiel-Demby 2008;Richter 2009 |
| Saquinavir (+ Ritonavir inhibitor) | 8.19E-08 | 5.23E-06 |  | 4.68E-06 | -5.33 | Alsenz 2003 |
| Quercetin | 1.36E-07 | 7.50E-06 |  | 9.25E-05 | -4.03 | Hayeshi 2006 |
| Cefamandole | 1.01E-07 | 6.19E-06 |  | 2.69E-07 | -6.50 | Raeissi 1999;Raeissi 1999 |
| Cefoperazone | 8.42E-08 | 5.32E-06 |  | 2.92E-07 | -6.53 | Raeissi 1999 |
| Cefoxitin | 1.06E-07 | 6.41E-06 |  | 1.56E-07 | -6.81 | Raeissi 1999 |
| Cimetidine | 1.57E-07 | 8.14E-06 | 2.31E-06 | 9.60E-07 | -5.79 | Richter 2009 |
| Cimetidine | 1.57E-07 | 8.14E-06 | 1.55E-06 |  | -5.81 | Garberg 2005 |
| Doxorubicin | 9.19E-08 | 5.75E-06 | 1.05E-06 | 1.92E-06 | -5.83 | Richter 2009 |
| Erythromycin | 7.85E-08 | 5.02E-06 |  | 2.31E-06 | -5.64 | Richter 2009 |
| Mitoxantrone | 1.03E-07 | 6.30E-06 | 3.06E-06 | 1.50E-07 | -5.79 | Richter 2009 |
| netivudine | 1.44E-07 | 7.74E-06 | 1.40E-06 | 6.80E-07 | -5.98 | Irvine 1999 |
| Sucrose | 1.24E-07 | 7.09E-06 | 3.30E-07 | 9.50E-07 | -6.19 | Garberg 2005 |
| terbutaline | 1.73E-07 | 8.57E-06 | 1.00E-06 | 4.10E-07 | -5.85 | Irvine 1999;Richter 2009 |
| bupropion | 1.64E-07 | 8.33E-06 | 1.30E-04 | 1.50E-04 | -3.96 | Irvine 1999;Summerfield 2007 |
| Carbamazepine | 1.66E-07 | 8.39E-06 | 2.35E-05 |  | -4.40 | Wang 2005;Thiel-Demby 2008;Summerfield 2007 |
| Chlorpromazine | 1.30E-07 | 7.32E-06 | 7.40E-06 |  | -5.13 | Summerfield 2007 |
| Diazepam | 1.42E-07 | 7.71E-06 | 4.64E-05 |  | -4.33 | Summerfield 2007 |
| Diazepam | 1.42E-07 | 7.71E-06 | 5.51E-05 | 4.82E-05 | -4.29 | Garberg 2005 |
| Flavone | 1.75E-07 | 8.62E-06 |  | 3.02E-05 | -4.52 | Hayeshi 2006 |
| guanabenz | 1.70E-07 | 8.47E-06 | 1.90E-04 | 1.00E-04 | -3.85 | Irvine 1999;Richter 2009 |
| Haloperidol | 1.15E-07 | 6.80E-06 | 2.86E-05 |  | -4.54 | Summerfield 2007 |
| Loxapine | 1.27E-07 | 7.23E-06 | 1.82E-05 |  | -4.74 | Summerfield 2007 |
| Midazolam | 1.28E-07 | 7.25E-06 | 5.41E-05 | 2.12E-04 | -3.88 | Richter 2009 |
| progesterone | 1.31E-07 | 7.37E-06 | 1.60E-04 | 9.80E-05 | -3.89 | Irvine 1999 |
| Quetiapine | 1.14E-07 | 6.73E-06 | 3.30E-05 |  | -4.48 | Summerfield 2007 |
| Testosterone | 1.40E-07 | 7.66E-06 |  | 3.93E-05 | -3.92 | Alsenz 2003;Irvine 1999;Richter 2009 |
| Ziprasidone | 1.08E-07 | 6.51E-06 | 1.17E-05 |  | -4.93 | Summerfield 2007 |
| Terfenadine | 9.90E-08 | 6.13E-06 | 1.95E-06 |  | -5.71 | Obradovic 2007 |
| Ritonavir | 7.93E-08 | 5.06E-06 | 3.20E-05 | 9.46E-05 | -4.20 | Richter 2009 |
| acrivastine | 1.21E-07 | 7.03E-06 | 2.20E-06 | 1.90E-06 | -5.69 | Irvine 1999 |
| Alanine | 4.50E-07 | 1.30E-05 | 3.90E-06 | 3.85E-06 | -5.41 | Garberg 2005 |
| amoxicillin | 1.18E-07 | 6.88E-06 | 1.00E-06 |  | -6.38 | Thiel-Demby 2008;Irvine 1999 |
| AZT | 1.05E-07 | 6.36E-06 | 4.28E-06 |  | -5.37 | Garberg 2005 |
| bretylium |  | 8.28E-06 | 1.40E-06 | 1.10E-06 | -5.90 | Irvine 1999 |
| Cefaclor | 1.18E-07 | 6.86E-06 |  | 5.29E-07 | -6.28 | Raeissi 1999 |
| Cefadroxil | 1.18E-07 | 6.90E-06 |  | 4.80E-07 | -6.32 | Raeissi 1999 |
| cefatrizine | 1.01E-07 | 6.19E-06 | 2.50E-06 | 7.60E-07 | -5.79 | Irvine 1999 |
| Cefazolin | 1.03E-07 | 6.23E-06 |  | 4.17E-07 | -6.38 | Raeissi 1999 |
| Cefsulodine |  | 5.80E-06 |  | 1.20E-07 | -6.92 | Raeissi 1999 |
| Ceftazidime |  | 5.74E-06 |  | 2.46E-07 | -6.61 | Raeissi 1999 |
| Ceftriaxon | 9.13E-08 | 5.70E-06 | 2.09E-06 | 2.61E-06 | -5.63 | Richter 2009 |
| Cephalexin | 1.22E-07 | 7.04E-06 |  | 2.73E-07 | -6.47 | Raeissi 1999;Irvine 1999 |
| Cephaloglycin | 1.10E-07 | 6.57E-06 |  | 2.67E-07 | -6.57 | Raeissi 1999 |
| Cephaloridine | | 6.49E-06 |  | 3.81E-07 | -6.42 | Raeissi 1999 |
| Cephradine | 1.22E-07 | 7.02E-06 |  | 4.35E-07 | -6.36 | Raeissi 1999 |
| Cetirizine | 1.13E-07 | 6.69E-06 | 2.38E-06 |  | -5.62 | Obradovic 2007 |
| gabapentin | 2.24E-07 | 9.70E-06 | 3.60E-07 | 1.00E-08 | -6.59 | Irvine 1999;Summerfield 2007 |
| Gatifloxacin | 1.16E-07 | 6.80E-06 | 1.03E-06 |  | -5.99 | Robertson 2005 |
| L-Dopa | 1.93E-07 | 9.10E-06 | 1.78E-06 |  | -5.75 | Garberg 2005 |
| Leucine | 2.93E-07 | 1.09E-05 | 3.95E-06 | 1.02E-05 | -5.15 | Garberg 2005 |
| lisinopril | 1.09E-07 | 6.57E-06 | 5.00E-07 |  | -6.52 | Thiel-Demby 2008;Irvine 1999 |
| Lomefloxacin | 1.21E-07 | 7.01E-06 | 6.60E-07 |  | -6.18 | Robertson 2005 |
| loracarbef-D | 1.22E-07 | 7.02E-06 | 9.10E-07 | 2.40E-07 | -6.24 | Irvine 1999 |
| Methotrexate | 1.02E-07 | 6.24E-06 | 1.55E-07 |  | -5.57 | Wang 2005;Richter 2009 |
| Moxifloxacin | 1.11E-07 | 6.60E-06 | 3.52E-06 |  | -5.45 | Robertson 2005 |
| olsalazine | 1.36E-07 | 7.50E-06 | 4.80E-08 | 1.60E-08 | -7.49 | Irvine 1999 |
| Phenylalanine | 2.32E-07 | 9.86E-06 |  | 1.49E-05 | -4.83 | Alsenz 2003 |
| Salicylic acid | 2.79E-07 | 1.07E-05 | 1.00E-05 | 1.30E-05 | -4.94 | Irvine 1999 |
| Sulfasalazine | 1.11E-07 | 6.62E-06 | 1.25E-07 |  | -6.11 | Wang 2005;Irvine 1999;Richter 2009 |
| Taurocholic acid | 9.42E-08 | 5.89E-06 |  | 1.28E-06 | -5.89 | Alsenz 2003 |

Table 4. Predicted resistances and resulting overall permeability

| **name** | **R_para_ (pH 7.4)** | **R_UWL_** | **R_cytosol_** | **R_filter_** | **R_mem_ apical (pH 7.4)** | **R_mem_ basal (pH 7.4)** | **R_lateral_ (30°C)** | **log P_app_ (calc)** |
| --- | --- | --- | --- | --- | --- | --- | --- | --- |
| acebutolol | 5.85E+05 | 2.47E+04 | 8.40E+02 | 1.11E+03 | 1.28E+04 | 3.06E+05 | 9.48E+07 | 4.54E-06 |
| acetylsalicylic acid | 7.03E+05 | 2.47E+04 | 6.33E+02 | 8.39E+02 | 1.11E+04 | 2.66E+05 | 2.14E+09 | 4.67E-06 |
| Amiloride | 5.68E+05 | 2.47E+04 | 7.06E+02 | 9.37E+02 | 1.11E+04 | 2.67E+05 | 3.65E+06 | 5.24E-06 |
| Fluvastatin | 1.53E+06 | 2.47E+04 | 9.20E+02 | 1.22E+03 | 1.80E+03 | 4.31E+04 | 3.68E+06 | 1.48E-05 |
| Genistein | 1.01E+06 | 1.28E+04 | 7.60E+02 | 1.01E+03 | 6.40E+02 | 1.54E+04 | 1.96E+05 | 3.84E-05 |
| hydrocortisone | 9.29E+05 | 2.47E+04 | 8.69E+02 | 1.15E+03 | 2.42E+03 | 5.80E+04 | 6.46E+06 | 1.27E-05 |
| Levofloxacin | 1.31E+06 | 2.47E+04 | 8.67E+02 | 1.15E+03 | 1.73E+04 | 4.15E+05 | 7.44E+08 | 2.93E-06 |
| methylprednisolone | 9.65E+05 | 2.47E+04 | 8.82E+02 | 1.17E+03 | 1.42E+03 | 3.40E+04 | 4.23E+06 | 1.73E-05 |
| Ofloxacin | 1.31E+06 | 2.47E+04 | 8.67E+02 | 1.15E+03 | 1.73E+04 | 4.15E+05 | 7.44E+08 | 2.93E-06 |
| practolol | 4.45E+05 | 2.47E+04 | 7.55E+02 | 1.00E+03 | 4.42E+03 | 1.06E+05 | 5.43E+08 | 9.43E-06 |
| Ranitidine | 5.08E+05 | 2.47E+04 | 8.14E+02 | 1.08E+03 | 8.18E+03 | 1.96E+05 | 3.28E+09 | 6.20E-06 |
| sumatriptan | 5.01E+05 | 2.47E+04 | 7.92E+02 | 1.05E+03 | 2.86E+03 | 6.85E+04 | 2.40E+08 | 1.21E-05 |
| Urea | 1.93E+05 | 1.63E+04 | 3.85E+02 | 5.10E+02 | 1.63E+03 | 3.92E+04 | 3.19E+07 | 2.20E-05 |
| acetaminophen | 3.85E+05 | 2.47E+04 | 5.84E+02 | 7.75E+02 | 2.24E+02 | 5.38E+03 | 1.10E+06 | 3.49E-05 |
| alprenolol | 4.14E+05 | 2.47E+04 | 7.33E+02 | 9.72E+02 | 5.90E-03 | 1.42E-01 | 1.60E+06 | 4.07E-05 |
| Amantadine | 2.51E+05 | 2.47E+04 | 5.85E+02 | 7.75E+02 | 4.13E-03 | 9.92E-02 | 3.70E+07 | 4.20E-05 |
| Amitriptyline | 4.66E+05 | 2.47E+04 | 7.69E+02 | 1.02E+03 | 4.90E-05 | 1.18E-03 | 4.35E+04 | 5.42E-05 |
| Amodiaquine | 6.05E+05 | 1.28E+04 | 8.61E+02 | 1.14E+03 | 1.09E-01 | 2.62E+00 | 6.10E+04 | 8.25E-05 |
| Antipyrine | 4.68E+05 | 2.47E+04 | 6.45E+02 | 8.56E+02 | 8.35E-01 | 2.01E+01 | 1.08E+07 | 4.02E-05 |
| Antipyrine | 4.68E+05 | 1.63E+04 | 6.45E+02 | 8.56E+02 | 8.35E-01 | 2.01E+01 | 1.08E+07 | 5.82E-05 |
| Caffeine | 4.82E+05 | 2.47E+04 | 6.55E+02 | 8.68E+02 | 7.87E-01 | 1.89E+01 | 7.73E+06 | 4.02E-05 |
| Caffeine | 4.82E+05 | 1.63E+04 | 6.55E+02 | 8.68E+02 | 7.87E-01 | 1.89E+01 | 7.73E+06 | 5.81E-05 |
| Chloramphenicol | 8.29E+05 | 2.47E+04 | 8.25E+02 | 1.09E+03 | 3.35E+01 | 8.03E+02 | 1.35E+06 | 3.83E-05 |
| Chloroquine | 4.67E+05 | 2.47E+04 | 8.21E+02 | 1.09E+03 | 3.57E-04 | 8.56E-03 | 1.58E+05 | 4.50E-05 |
| Chloroquine | 4.67E+05 | 1.28E+04 | 8.21E+02 | 1.09E+03 | 3.57E-04 | 8.56E-03 | 1.58E+05 | 7.59E-05 |
| Clemastine | 6.01E+05 | 2.47E+04 | 8.48E+02 | 1.12E+03 | 1.41E-04 | 3.39E-03 | 6.41E+04 | 5.02E-05 |
| Clonidine | 3.98E+05 | 2.47E+04 | 7.07E+02 | 9.38E+02 | 1.24E+00 | 2.99E+01 | 1.09E+07 | 4.03E-05 |
| Clozapine | 6.77E+05 | 2.47E+04 | 8.29E+02 | 1.10E+03 | 2.35E-03 | 5.63E-02 | 4.44E+04 | 5.32E-05 |
| corticosterone | 8.82E+05 | 2.47E+04 | 8.51E+02 | 1.13E+03 | 4.24E+00 | 1.02E+02 | 3.81E+05 | 4.08E-05 |
| Desipramine | 4.45E+05 | 2.47E+04 | 7.55E+02 | 1.00E+03 | 1.06E-03 | 2.53E-02 | 2.02E+05 | 4.43E-05 |
| dexamethasone | 1.02E+06 | 2.47E+04 | 9.00E+02 | 1.19E+03 | 1.52E+01 | 3.64E+02 | 1.45E+05 | 4.36E-05 |
| Doxepin | 4.70E+05 | 2.47E+04 | 7.72E+02 | 1.02E+03 | 2.88E-03 | 6.92E-02 | 6.83E+05 | 4.11E-05 |
| Ethosuximide | 3.62E+05 | 2.47E+04 | 5.67E+02 | 7.52E+02 | 1.88E-01 | 4.50E+00 | 2.09E+05 | 4.52E-05 |
| Fluoxetine | 5.29E+05 | 2.47E+04 | 8.08E+02 | 1.07E+03 | 4.35E-04 | 1.04E-02 | 2.33E+05 | 4.32E-05 |
| fluparoxan | 3.20E+05 | 2.47E+04 | 6.56E+02 | 8.70E+02 | 4.29E+01 | 1.03E+03 | 5.39E+08 | 3.95E-05 |
| Gly-Pro | 3.91E+05 | 2.47E+04 | 5.90E+02 | 7.82E+02 | 3.27E+02 | 7.85E+03 | 2.49E+08 | 3.16E-05 |
| Hydroxyzine | 7.82E+05 | 2.47E+04 | 8.82E+02 | 1.17E+03 | 1.11E-03 | 2.65E-02 | 3.11E+04 | 5.61E-05 |
| Imipramine | 4.73E+05 | 2.47E+04 | 7.73E+02 | 1.03E+03 | 2.49E-04 | 5.98E-03 | 1.30E+05 | 4.61E-05 |
| Isocarboxazid | 5.72E+05 | 2.47E+04 | 7.09E+02 | 9.40E+02 | 3.02E-02 | 7.25E-01 | 1.28E+05 | 4.61E-05 |
| ketoprofen | 9.57E+05 | 2.47E+04 | 7.40E+02 | 9.81E+02 | 2.60E+01 | 6.24E+02 | 1.97E+07 | 3.80E-05 |
| labetalol | 6.02E+05 | 2.47E+04 | 8.31E+02 | 1.10E+03 | 2.76E+02 | 6.63E+03 | 1.13E+06 | 3.22E-05 |
| lamotrigine | 6.26E+05 | 2.47E+04 | 7.42E+02 | 9.84E+02 | 1.65E-02 | 3.97E-01 | 3.43E+04 | 5.60E-05 |
| Lansoprazole | 9.52E+05 | 2.47E+04 | 8.76E+02 | 1.16E+03 | 9.84E-02 | 2.36E+00 | 7.78E+04 | 4.80E-05 |
| metoprolol | 4.47E+05 | 2.47E+04 | 7.57E+02 | 1.00E+03 | 4.04E+00 | 9.70E+01 | 1.03E+08 | 3.97E-05 |
| minoxidil | 5.18E+05 | 2.47E+04 | 6.77E+02 | 8.98E+02 | 2.88E+02 | 6.92E+03 | 1.01E+07 | 3.18E-05 |
| Mirtazapine | 6.11E+05 | 2.47E+04 | 7.54E+02 | 1.00E+03 | 3.97E-04 | 9.53E-03 | 3.31E+04 | 5.63E-05 |
| Morphine | 4.83E+05 | 1.63E+04 | 7.79E+02 | 1.03E+03 | 5.93E+02 | 1.42E+04 | 1.36E+08 | 3.24E-05 |
| naproxen | 8.73E+05 | 2.47E+04 | 7.07E+02 | 9.38E+02 | 5.75E+00 | 1.38E+02 | 5.46E+06 | 3.90E-05 |
| Nicotine | 2.73E+05 | 1.63E+04 | 6.03E+02 | 8.00E+02 | 9.74E-02 | 2.34E+00 | 1.48E+07 | 5.99E-05 |
| Omeprazole | 8.82E+05 | 2.47E+04 | 8.50E+02 | 1.13E+03 | 5.24E+00 | 1.26E+02 | 4.55E+05 | 4.04E-05 |
| ondansetron | 5.99E+05 | 2.47E+04 | 7.89E+02 | 1.05E+03 | 3.56E-02 | 8.55E-01 | 2.65E+05 | 4.27E-05 |
| oxyprenolol | 3.13E+05 | 2.47E+04 | 5.23E+02 | 6.93E+02 | 1.24E+01 | 2.97E+02 | 5.93E+05 | 4.27E-05 |
| Pantoprazole | 9.97E+05 | 2.47E+04 | 8.91E+02 | 1.18E+03 | 5.54E+01 | 1.33E+03 | 3.27E+06 | 3.68E-05 |
| Pemoline | 4.40E+05 | 2.47E+04 | 6.26E+02 | 8.31E+02 | 1.24E+01 | 2.97E+02 | 1.99E+07 | 3.99E-05 |
| Pergolide | 5.40E+05 | 2.47E+04 | 8.14E+02 | 1.08E+03 | 2.08E-01 | 5.00E+00 | 3.04E+05 | 4.23E-05 |
| Phenelzine | 3.49E+05 | 2.47E+04 | 5.57E+02 | 7.39E+02 | 1.94E-03 | 4.65E-02 | 3.57E+04 | 5.74E-05 |
| phenytoin | 6.42E+05 | 2.47E+04 | 7.37E+02 | 9.78E+02 | 7.32E-01 | 1.76E+01 | 2.71E+04 | 5.82E-05 |
| Phenytoin | 6.42E+05 | 1.63E+04 | 7.37E+02 | 9.78E+02 | 7.32E-01 | 1.76E+01 | 2.71E+04 | 7.94E-05 |
| pindolol | 4.12E+05 | 2.47E+04 | 7.32E+02 | 9.71E+02 | 9.40E+00 | 2.26E+02 | 4.19E+07 | 3.98E-05 |
| Praziquantel | 7.86E+05 | 1.28E+04 | 8.12E+02 | 1.08E+03 | 3.16E-04 | 7.58E-03 | 1.63E+04 | 1.02E-04 |
| Prazosine | 8.39E+05 | 2.47E+04 | 8.91E+02 | 1.18E+03 | 2.14E-01 | 5.13E+00 | 2.20E+05 | 4.25E-05 |
| propranolol | 4.32E+05 | 2.47E+04 | 7.46E+02 | 9.90E+02 | 3.90E-01 | 9.36E+00 | 1.93E+06 | 4.05E-05 |
| propylthiouracil | 5.31E+05 | 2.47E+04 | 6.17E+02 | 8.18E+02 | 3.54E+00 | 8.50E+01 | 5.35E+05 | 4.17E-05 |
| Pumafentrine | 1.66E+06 | 2.47E+04 | 9.84E+02 | 1.31E+03 | 1.12E-03 | 2.68E-02 | 6.97E+04 | 4.80E-05 |
| Quinidine | 5.63E+05 | 2.47E+04 | 8.26E+02 | 1.10E+03 | 2.85E-01 | 6.83E+00 | 1.63E+06 | 3.98E-05 |
| Quinine | 5.63E+05 | 1.28E+04 | 8.26E+02 | 1.10E+03 | 2.85E-01 | 6.83E+00 | 1.63E+06 | 7.03E-05 |
| Selegiline | 3.12E+05 | 2.47E+04 | 6.44E+02 | 8.54E+02 | 1.66E-03 | 3.97E-02 | 5.06E+05 | 4.30E-05 |
| sotalol | 4.57E+05 | 2.47E+04 | 7.63E+02 | 1.01E+03 | 7.49E+02 | 1.80E+04 | 2.13E+08 | 2.42E-05 |
| Tacrine | 3.28E+05 | 2.47E+04 | 6.61E+02 | 8.77E+02 | 1.39E-03 | 3.33E-02 | 5.03E+04 | 5.41E-05 |
| Temazepam | 7.54E+05 | 2.47E+04 | 7.98E+02 | 1.06E+03 | 1.23E-02 | 2.95E-01 | 3.98E+04 | 5.41E-05 |
| theophylline | 5.00E+05 | 2.47E+04 | 6.33E+02 | 8.39E+02 | 3.18E+02 | 7.64E+03 | 3.47E+07 | 3.12E-05 |
| Thiabendazole | 4.99E+05 | 1.28E+04 | 6.65E+02 | 8.82E+02 | 9.81E-02 | 2.35E+00 | 4.88E+04 | 8.77E-05 |
| timolol | 5.43E+05 | 2.47E+04 | 8.17E+02 | 1.08E+03 | 1.13E+02 | 2.71E+03 | 2.21E+09 | 3.57E-05 |
| Tolafentrine | 1.12E+06 | 2.47E+04 | 1.01E+03 | 1.34E+03 | 2.28E-02 | 5.48E-01 | 6.63E+04 | 4.86E-05 |
| Trazodone | 8.35E+05 | 2.47E+04 | 8.79E+02 | 1.17E+03 | 4.13E-03 | 9.91E-02 | 5.96E+04 | 5.02E-05 |
| trimethoprim | 8.25E+05 | 2.47E+04 | 7.86E+02 | 1.04E+03 | 3.79E+01 | 9.08E+02 | 1.82E+06 | 3.81E-05 |
| verapamil | 8.60E+05 | 2.47E+04 | 9.62E+02 | 1.28E+03 | 1.36E-01 | 3.27E+00 | 3.69E+06 | 3.85E-05 |
| Verapamil | 8.60E+05 | 1.63E+04 | 9.62E+02 | 1.28E+03 | 1.36E-01 | 3.27E+00 | 3.69E+06 | 5.53E-05 |
| warfarin | 1.14E+06 | 2.47E+04 | 8.07E+02 | 1.07E+03 | 7.95E+00 | 1.91E+02 | 6.66E+06 | 3.83E-05 |
| Warfarin | 1.14E+06 | 1.63E+04 | 8.07E+02 | 1.07E+03 | 7.95E+00 | 1.91E+02 | 6.66E+06 | 5.54E-05 |
| Zidovudine | 6.65E+05 | 2.47E+04 | 7.57E+02 | 1.00E+03 | 7.43E+01 | 1.78E+03 | 1.58E+07 | 3.68E-05 |
| bupropion | 4.14E+05 | 2.47E+04 | 7.20E+02 | 9.55E+02 | 2.81E-06 | 6.75E-05 | 3.29E+03 | 7.46E-05 |
| Carbamazepine | 5.85E+05 | 2.47E+04 | 7.16E+02 | 9.49E+02 | 1.97E-03 | 4.72E-02 | 4.17E+03 | 7.30E-05 |
| Chlorpromazine | 5.50E+05 | 2.47E+04 | 8.20E+02 | 1.09E+03 | 9.41E-06 | 2.26E-04 | 6.85E+03 | 6.99E-05 |
| Diazepam | 7.10E+05 | 2.47E+04 | 7.79E+02 | 1.03E+03 | 9.54E-05 | 2.29E-03 | 1.39E+04 | 6.43E-05 |
| Diazepam | 7.10E+05 | 1.63E+04 | 7.79E+02 | 1.03E+03 | 9.54E-05 | 2.29E-03 | 1.39E+04 | 8.86E-05 |
| Flavone | 5.50E+05 | 1.28E+04 | 6.96E+02 | 9.23E+02 | 2.63E-05 | 6.32E-04 | 1.95E+03 | 1.35E-04 |
| guanabenz | 3.99E+05 | 2.47E+04 | 7.08E+02 | 9.39E+02 | 3.10E-03 | 7.44E-02 | 9.70E+03 | 6.85E-05 |
| Haloperidol | 7.11E+05 | 2.47E+04 | 8.83E+02 | 1.17E+03 | 1.55E-03 | 3.73E-02 | 1.97E+04 | 6.06E-05 |
| Loxapine | 7.06E+05 | 2.47E+04 | 8.30E+02 | 1.10E+03 | 4.27E-05 | 1.02E-03 | 1.05E+04 | 6.64E-05 |
| Midazolam | 7.77E+05 | 2.47E+04 | 8.28E+02 | 1.10E+03 | 1.36E-04 | 3.26E-03 | 9.01E+03 | 6.75E-05 |
| progesterone | 7.92E+05 | 2.47E+04 | 8.14E+02 | 1.08E+03 | 1.33E-04 | 3.19E-03 | 3.81E+03 | 7.25E-05 |
| Quetiapine | 8.70E+05 | 2.47E+04 | 8.91E+02 | 1.18E+03 | 1.40E-03 | 3.35E-02 | 1.16E+04 | 6.51E-05 |
| Testosterone | 7.20E+05 | 2.47E+04 | 7.83E+02 | 1.04E+03 | 2.32E-03 | 5.56E-02 | 8.23E+03 | 6.84E-05 |
| Ziprasidone | 9.48E+05 | 2.47E+04 | 9.21E+02 | 1.22E+03 | 1.52E-03 | 3.65E-02 | 1.40E+03 | 7.48E-05 |
| Saquinavir (+ Ritonavir inhibitor) | 1.51E+06 | 2.47E+04 | 1.15E+03 | 1.52E+03 | 1.36E+03 | 3.27E+04 | 7.62E+03 | 4.64E-05 |
| Quercetin | 1.18E+06 | 1.28E+04 | 8.00E+02 | 1.06E+03 | 1.40E+06 | 3.35E+07 | 6.82E+05 | 2.30E-06 |
| Terfenadine | 9.09E+05 | 2.47E+04 | 9.79E+02 | 1.30E+03 | 4.32E-04 | 1.04E-02 | 5.97E+02 | 7.56E-05 |
| Ritonavir | 2.14E+06 | 2.47E+04 | 1.19E+03 | 1.57E+03 | 1.79E-03 | 4.30E-02 | 9.41E+00 | 7.48E-05 |
| acyclovir | 6.01E+05 | 2.47E+04 | 7.00E+02 | 9.29E+02 | 1.90E+08 | 4.56E+09 | 1.67E+10 | 1.60E-06 |
| Atenolol | 4.45E+05 | 2.47E+04 | 7.55E+02 | 1.00E+03 | 5.19E+04 | 1.24E+06 | 1.95E+09 | 2.88E-06 |
| Cefotaxime | 1.70E+06 | 2.47E+04 | 9.63E+02 | 1.28E+03 | 9.52E+08 | 2.28E+10 | 1.26E+11 | 5.79E-07 |
| Cefuroxime | 1.58E+06 | 2.47E+04 | 9.33E+02 | 1.24E+03 | 4.27E+08 | 1.03E+10 | 5.99E+10 | 6.22E-07 |
| Cephalothin | 1.48E+06 | 2.47E+04 | 9.05E+02 | 1.20E+03 | 1.92E+05 | 4.61E+06 | 2.24E+09 | 8.73E-07 |
| Cephapirin | 1.58E+06 | 2.47E+04 | 9.32E+02 | 1.24E+03 | 6.31E+06 | 1.51E+08 | 2.40E+10 | 6.31E-07 |
| Chlorothiazide | 7.45E+05 | 2.47E+04 | 7.92E+02 | 1.05E+03 | 2.32E+05 | 5.58E+06 | 4.35E+08 | 1.47E-06 |
| Ciprofloxacin | 8.44E+05 | 2.47E+04 | 8.34E+02 | 1.11E+03 | 5.42E+05 | 1.30E+07 | 3.73E+09 | 1.22E-06 |
| Furosemide | 1.23E+06 | 2.47E+04 | 8.33E+02 | 1.11E+03 | 2.60E+07 | 6.25E+08 | 4.76E+09 | 7.96E-07 |
| Glycerol | 2.58E+05 | 1.63E+04 | 4.67E+02 | 6.19E+02 | 7.33E+04 | 1.76E+06 | 5.39E+08 | 4.18E-06 |
| hydrochlorothiazide | 7.50E+05 | 2.47E+04 | 7.95E+02 | 1.05E+03 | 1.58E+06 | 3.79E+07 | 9.08E+08 | 1.31E-06 |
| Lactic acid | 4.12E+05 | 1.63E+04 | 4.62E+02 | 6.13E+02 | 3.17E+05 | 7.60E+06 | 4.30E+10 | 2.46E-06 |
| Mannitol | 4.54E+05 | 2.47E+04 | 6.36E+02 | 8.44E+02 | 6.89E+06 | 1.65E+08 | 4.89E+09 | 2.09E-06 |
| nadolol | 5.29E+05 | 2.47E+04 | 8.08E+02 | 1.07E+03 | 3.22E+04 | 7.73E+05 | 2.16E+09 | 3.01E-06 |
| Norfloxacin | 8.10E+05 | 2.47E+04 | 8.20E+02 | 1.09E+03 | 4.25E+05 | 1.02E+07 | 7.82E+09 | 1.29E-06 |
| penicillin v | 1.30E+06 | 2.47E+04 | 8.55E+02 | 1.13E+03 | 1.37E+05 | 3.30E+06 | 3.77E+09 | 1.04E-06 |
| sulpiride | 6.12E+05 | 2.47E+04 | 8.45E+02 | 1.12E+03 | 5.78E+04 | 1.39E+06 | 9.86E+07 | 2.26E-06 |
| Cefamandole | 1.73E+06 | 2.47E+04 | 9.70E+02 | 1.29E+03 | 1.40E+10 | 3.35E+11 | 2.30E+11 | 5.70E-07 |
| Cefoperazone | 2.44E+06 | 2.47E+04 | 1.13E+03 | 1.50E+03 | 1.66E+14 | 3.99E+15 | 4.61E+12 | 4.06E-07 |
| Cefoxitin | 1.59E+06 | 2.47E+04 | 9.36E+02 | 1.24E+03 | 3.16E+09 | 7.59E+10 | 2.76E+10 | 6.17E-07 |
| Cimetidine | 5.91E+05 | 2.47E+04 | 7.37E+02 | 9.78E+02 | 5.46E+07 | 1.31E+09 | 2.35E+08 | 1.63E-06 |
| Cimetidine | 5.91E+05 | 1.63E+04 | 7.37E+02 | 9.78E+02 | 5.46E+07 | 1.31E+09 | 2.35E+08 | 1.65E-06 |
| Doxorubicin | 1.16E+06 | 2.47E+04 | 1.04E+03 | 1.38E+03 | 1.59E+10 | 3.81E+11 | 4.66E+08 | 8.43E-07 |
| Erythromycin | 1.73E+06 | 2.47E+04 | 1.20E+03 | 1.59E+03 | 1.49E+06 | 3.57E+07 | 2.52E+07 | 6.37E-07 |
| Mitoxantrone | 8.35E+05 | 2.47E+04 | 9.53E+02 | 1.26E+03 | 5.21E+15 | 1.25E+17 | 1.66E+12 | 1.16E-06 |
| netivudine | 7.06E+05 | 2.47E+04 | 7.76E+02 | 1.03E+03 | 7.96E+06 | 1.91E+08 | 1.86E+08 | 1.38E-06 |
| Sucrose | 8.70E+05 | 1.63E+04 | 8.46E+02 | 1.12E+03 | 9.69E+13 | 2.33E+15 | 4.64E+10 | 1.13E-06 |
| terbutaline | 3.75E+05 | 2.47E+04 | 7.00E+02 | 9.29E+02 | 3.45E+06 | 8.27E+07 | 4.65E+07 | 2.53E-06 |
| acrivastine | 9.04E+05 | 2.47E+04 | 8.53E+02 | 1.13E+03 | NA | NA | NA | 1.08E-06 |
| Alanine | 2.53E+05 | 1.63E+04 | 4.60E+02 | 6.10E+02 | NA | NA | NA | 3.71E-06 |
| amoxicillin | 1.16E+06 | 2.47E+04 | 8.72E+02 | 1.16E+03 | NA | NA | NA | 8.44E-07 |
| AZT | 2.15E+06 | 1.63E+04 | 9.44E+02 | 1.25E+03 | NA | NA | NA | 4.60E-07 |
| bretylium | 4.02E+05 | 6.50E+04 | 7.25E+02 | 9.61E+02 | NA | NA | NA | 2.14E-06 |
| Cefaclor | 1.16E+06 | 2.47E+04 | 8.74E+02 | 1.16E+03 | NA | NA | NA | 8.44E-07 |
| Cefadroxil | 1.15E+06 | 2.47E+04 | 8.70E+02 | 1.15E+03 | NA | NA | NA | 8.49E-07 |
| cefatrizine | 1.50E+06 | 2.47E+04 | 9.70E+02 | 1.29E+03 | NA | NA | NA | 6.55E-07 |
| Cefazolin | 1.70E+06 | 2.47E+04 | 9.62E+02 | 1.28E+03 | NA | NA | NA | 5.80E-07 |
| Cefsulodine | 2.00E+06 | 2.47E+04 | 1.03E+03 | 1.37E+03 | NA | NA | NA | 4.94E-07 |
| Ceftazidime | 2.05E+06 | 2.47E+04 | 1.05E+03 | 1.39E+03 | NA | NA | NA | 4.81E-07 |
| Ceftriaxon | 2.62E+06 | 2.47E+04 | 1.05E+03 | 1.40E+03 | NA | NA | NA | 3.78E-07 |
| Cephalexin | 1.09E+06 | 2.47E+04 | 8.52E+02 | 1.13E+03 | NA | NA | NA | 8.96E-07 |
| Cephaloglycin | 1.29E+06 | 2.47E+04 | 9.14E+02 | 1.21E+03 | NA | NA | NA | 7.61E-07 |
| Cephaloridine | 1.09E+06 | 2.47E+04 | 9.24E+02 | 1.23E+03 | NA | NA | NA | 8.96E-07 |
| Cephradine | 1.02E+06 | 2.47E+04 | 8.54E+02 | 1.13E+03 | NA | NA | NA | 9.60E-07 |
| Cetirizine | 1.18E+06 | 2.47E+04 | 8.97E+02 | 1.19E+03 | NA | NA | NA | 8.27E-07 |
| gabapentin | 4.29E+05 | 2.47E+04 | 6.18E+02 | 8.20E+02 | NA | NA | NA | 2.20E-06 |
| Gatifloxacin | 9.73E+05 | 2.47E+04 | 8.82E+02 | 1.17E+03 | NA | NA | NA | 1.00E-06 |
| L-Dopa | 4.93E+05 | 1.63E+04 | 6.59E+02 | 8.74E+02 | NA | NA | NA | 1.96E-06 |
| Leucine | 3.41E+05 | 1.63E+04 | 5.48E+02 | 7.27E+02 | NA | NA | NA | 2.79E-06 |
| lisinopril | 1.13E+06 | 2.47E+04 | 9.14E+02 | 1.21E+03 | NA | NA | NA | 8.69E-07 |
| Lomefloxacin | 9.04E+05 | 2.47E+04 | 8.56E+02 | 1.14E+03 | NA | NA | NA | 1.08E-06 |
| loracarbef-D | 1.10E+06 | 2.47E+04 | 8.55E+02 | 1.13E+03 | NA | NA | NA | 8.89E-07 |
| Methotrexate | 2.23E+06 | 2.47E+04 | 9.62E+02 | 1.28E+03 | NA | NA | NA | 4.43E-07 |
| Moxifloxacin | 1.04E+06 | 2.47E+04 | 9.10E+02 | 1.21E+03 | NA | NA | NA | 9.38E-07 |
| olsalazine | 1.60E+06 | 2.47E+04 | 8.00E+02 | 1.06E+03 | NA | NA | NA | 6.16E-07 |
| Phenylalanine | 4.17E+05 | 2.47E+04 | 6.08E+02 | 8.07E+02 | NA | NA | NA | 2.26E-06 |
| Salicylic acid | 5.65E+05 | 2.47E+04 | 5.61E+02 | 7.44E+02 | NA | NA | NA | 1.69E-06 |
| Sulfasalazine | 1.88E+06 | 2.47E+04 | 9.07E+02 | 1.20E+03 | NA | NA | NA | 5.24E-07 |
| Taurocholic acid | 1.93E+06 | 2.47E+04 | 1.02E+03 | 1.35E+03 | NA | NA | NA | 5.11E-07 |

Table 5. pp-LFER descriptors from UFZ-LSER database. As outlined in the paper the descriptors are experimentally determined descriptors taken from the literature, if available. Otherwise, they are predicted with the QSAR available from the same source [3].

| **name** | **E** | **S** | **A** | **B** | **V** | **L** |
| --- | --- | --- | --- | --- | --- | --- |
| acebutolol | 1.6 | 2.42 | 0.9 | 2.1 | 2.7556 |  |
| acetaminophen | 1.06 | 1.63 | 1.04 | 0.86 | 1.1724 | 6.43 |
| acetylsalicylic acid | 0.78 | 1.69 | 0.71 | 0.67 | 1.2879 | 6.279 |
| alprenolol | 1.25 | 1.03 | 0.1 | 1.25 | 2.1587 |  |
| Amantadine | -1.9 | 1.77 | -0.24 | -0.19 | 1.2916 | 5.682 |
| Amiloride | 2.4 | 2.38 | 1.1 | 1.39 | 1.5101 | 8.985 |
| Amitriptyline | 1.92 | 1.4 | 0 | 1.06 | 2.3996 | 11.31 |
| Amodiaquine | 2.56 | 2.23 | 0.65 | 1.4 | 2.7373 | 13.232 |
| Antipyrine | 1.3 | 1.83 | 0 | 1.37 | 1.4846 |  |
| Antipyrine | 1.3 | 1.83 | 0 | 1.37 | 1.4846 |  |
| Caffeine | 1.5 | 1.72 | 0.05 | 1.28 | 1.3632 | 7.352 |
| Caffeine | 1.5 | 1.72 | 0.05 | 1.28 | 1.3632 | 7.352 |
| Chloramphenicol | 1.89 | 1.82 | 0.75 | 1.74 | 2.0728 | 10.594 |
| Chloroquine | 1.69 | 1.47 | 0.12 | 1.14 | 2.6344 | 11.165 |
| Chloroquine | 1.69 | 1.47 | 0.12 | 1.14 | 2.6344 | 11.165 |
| Clemastine | 1.49 | 1.95 | 0 | 1.28 | 2.7646 | 11.376 |
| Clonidine | 1.56 | 1.58 | 0.16 | 1.27 | 1.5317 | 8.122 |
| Clozapine | 2.55 | 1.58 | 0.18 | 1.75 | 2.431 | 12.274 |
| corticosterone | 1.87 | 2.3 | 0.63 | 2.09 | 2.7389 | 12.804 |
| Desipramine | 1.62 | 1.82 | 0.09 | 0.91 | 2.2606 |  |
| dexamethasone | 2.04 | 3.51 | 0.71 | 1.92 | 2.9132 |  |
| Doxepin | 1.71 | 1.76 | 0 | 1.2 | 2.3174 | 9.602 |
| Ethosuximide | 0.58 | 1.17 | 0.59 | 0.6 | 1.1175 | 4.797 |
| Fluoxetine | 1 | 1.3 | 0.1 | 0.93 | 2.2403 |  |
| fluparoxan | 1.18 | 1.34 | 0.14 | 1.16 | 1.2977 | 6.129 |
| Fluvastatin | 2.68 | 2.93 | 1.07 | 1.97 | 3.1297 | 14.582 |
| Genistein | 2.34 | 2.05 | 1.39 | 1.12 | 1.8458 | 11.011 |
| Gly-Pro | 1 | 1.89 | 0.2 | 1.36 | 1.1087 | 5.821 |
| hydrocortisone | 2.03 | 3.49 | 0.71 | 1.9 | 2.7976 |  |
| Hydroxyzine | 2 | 2.21 | 0.1 | 1.89 | 2.9231 |  |
| Imipramine | 1.15 | 1.6 | 0 | 1.15 | 2.4015 |  |
| Isocarboxazid | 1.41 | 1.69 | 0.34 | 1.11 | 1.741 | 8.568 |
| ketoprofen | 1.65 | 2.26 | 0.55 | 0.89 | 1.9779 |  |
| labetalol | 2.27 | 1.99 | 1.18 | 1.64 | 2.6432 | 12.461 |
| lamotrigine | 2.27 | 2.03 | 0.35 | 0.96 | 1.6453 |  |
| Lansoprazole | 2.19 | 2.04 | 0.46 | 1.67 | 2.3696 | 11.829 |
| Levofloxacin | 2.39 | 2.76 | 0.46 | 2.28 | 2.5043 | 12.631 |
| methylprednisolone | 2.16 | 2.7 | 0.84 | 2.5 | 2.8955 | 13.882 |
| metoprolol | 1.17 | 1.33 | 0.17 | 1.76 | 2.2604 |  |
| minoxidil | 1.68 | 2.33 | 0.58 | 1.44 | 1.5882 | 8.474 |
| Mirtazapine | 2.09 | 1.73 | 0 | 1.37 | 2.1109 |  |
| Morphine | 2.12 | 1.88 | 0.49 | 1.83 | 2.0648 |  |
| naproxen | 1.51 | 1.98 | 0.6 | 0.68 | 1.7821 |  |
| Nicotine | 0.87 | 0.88 | 0 | 1.09 | 1.371 |  |
| Ofloxacin | 2.39 | 2.76 | 0.46 | 2.28 | 2.5043 | 12.631 |
| Omeprazole | 2.52 | 2.93 | 0.46 | 1.91 | 2.5161 | 13.369 |
| ondansetron | 2.48 | 2.82 | 0 | 1.56 | 2.2675 | 12.973 |
| oxyprenolol | 0.74 | 0.94 | 0.92 | 0.68 | 0.9462 | 4.252 |
| Pantoprazole | 2.4 | 2.83 | 0.46 | 2.09 | 2.4693 | 12.791 |
| Pemoline | 1.49 | 1.4 | 0.27 | 1.38 | 1.2615 | 6.382 |
| Pergolide | 2.44 | 2.24 | 0.46 | 1.44 | 2.5424 | 13.458 |
| Phenelzine | 0.98 | 0.86 | 0.35 | 0.6 | 1.1978 | 5.411 |
| phenytoin | 1.71 | 2.19 | 0.85 | 1 | 1.8693 |  |
| Phenytoin | 1.71 | 2.19 | 0.85 | 1 | 1.8693 |  |
| pindolol | 1.7 | 1.65 | 0.3 | 1.48 | 2.009 |  |
| practolol | 1.45 | 1.9 | 0.6 | 1.84 | 2.1763 |  |
| Praziquantel | 1.89 | 2.34 | 0 | 1.44 | 2.4533 | 12.048 |
| Prazosine | 2.82 | 2.54 | 0.27 | 2.09 | 2.7411 | 14.098 |
| propranolol | 1.84 | 1.43 | 0.44 | 1.31 | 2.148 |  |
| propylthiouracil | 1.22 | 1.28 | 0.76 | 0.86 | 1.2791 | 6.516 |
| Pumafentrine | 2.67 | 2.02 | 0 | 2.91 | 3.8918 | 16.606 |
| Quinidine | 2.47 | 1.23 | 0.37 | 1.97 | 2.5512 |  |
| Quinine | 2.47 | 1.23 | 0.37 | 1.97 | 2.5512 |  |
| Ranitidine | 1.55 | 2.75 | 0.18 | 2.28 | 2.3985 | 10.594 |
| Selegiline | 0.87 | 1.03 | 0.05 | 0.93 | 1.7165 | 6.342 |
| sotalol | 1.64 | 2.02 | 0.5 | 1.68 | 2.101 | 9.16 |
| sumatriptan | 1.83 | 2.3 | 0.57 | 1.85 | 2.2723 | 11.151 |
| Tacrine | 1.98 | 1.33 | 0.27 | 0.64 | 1.5991 | 8.629 |
| Temazepam | 2.52 | 1.87 | 0.31 | 1.49 | 2.1326 | 10.969 |
| theophylline | 1.5 | 1.6 | 0.54 | 1.34 | 1.2223 |  |
| Thiabendazole | 2.12 | 2.22 | 0.46 | 0.74 | 1.3967 | 8.236 |
| timolol | 1.78 | 1.77 | -0.03 | 2.22 | 2.3759 | 9.785 |
| Tolafentrine | 3.31 | 3.15 | 0.12 | 2.67 | 3.7785 | 18.286 |
| Trazodone | 2.77 | 2.61 | 0 | 1.91 | 2.7304 | 13.669 |
| trimethoprim | 2.52 | 2.81 | 0.5 | 1.76 | 2.1813 |  |
| Urea | 0.64 | 0.93 | 0.79 | 0.79 | 0.4648 | 2.461 |
| verapamil | 1.81 | 2.91 | 0 | 2.51 | 3.7861 |  |
| Verapamil | 1.81 | 2.91 | 0 | 2.51 | 3.7861 |  |
| warfarin | 2.01 | 2.27 | 0.41 | 1.57 | 2.3077 | 11.807 |
| Warfarin | 2.01 | 2.27 | 0.41 | 1.57 | 2.3077 | 11.807 |
| Zidovudine | 1.83 | 1.7 | 0.47 | 1.83 | 1.8192 |  |
| acyclovir | 2.03 | 2.2 | 0.99 | 2.34 | 1.5217 | 9.791 |
| Atenolol | 1.45 | 1.88 | 0.69 | 2 | 2.1763 |  |
| Cefotaxime | 3.61 | 2.94 | 1 | 2.9 | 2.9301 | 15.903 |
| Cefuroxime | 3.15 | 2.59 | 1.12 | 2.61 | 2.7255 | 14.466 |
| Cephalothin | 3.02 | 2.57 | 0.72 | 2.17 | 2.615 | 13.508 |
| Cephapirin | 2.99 | 2.97 | 0.72 | 2.46 | 2.8127 | 15.061 |
| Chlorothiazide | 2.26 | 3.01 | 0.63 | 1.93 | 1.6893 | 9.569 |
| Ciprofloxacin | 2.52 | 2.55 | 0.73 | 2.07 | 2.3047 | 11.947 |
| Furosemide | 1.97 | 2.82 | 1.16 | 1.71 | 2.1032 | 10.782 |
| Glycerol | 0.57 | 1.01 | 0.84 | 1.26 | 0.7074 | 3.706 |
| hydrochlorothiazide | 2.14 | 2.89 | 0.77 | 2.06 | 1.7323 | 9.878 |
| Lactic acid | 0.34 | 0.85 | 0.71 | 0.71 | 0.6644 | 2.942 |
| Mannitol | 0.84 | 2.26 | 0.86 | 1.79 | 1.3062 |  |
| nadolol | 1.79 | 2.05 | 0.58 | 2.29 | 2.4923 | 10.972 |
| Norfloxacin | 2.18 | 2.55 | 0.61 | 2.07 | 2.2724 | 11.397 |
| penicillin v | 2.56 | 2.23 | 0.72 | 1.97 | 2.4358 | 11.746 |
| sulpiride | 1.75 | 3.07 | 0.84 | 2.11 | 2.5306 | 11.576 |
| Saquinavir (+ Ritonavir inhibitor) | 4.02 | 4.75 | 1.43 | 3.57 | 5.2961 | 24.554 |
| Quercetin | 2.7 | 2.31 | 2.04 | 1.35 | 1.9632 | 12.296 |
| Cefamandole | 3.58 | 4.04 | 1.03 | 2.85 | 3.0427 | 17.099 |
| Cefoperazone | 4.74 | 6.31 | 1.17 | 4.09 | 4.2669 | 22.965 |
| Cefoxitin | 3.05 | 2.85 | 1.41 | 2.58 | 2.7735 | 14.331 |
| Cimetidine | 1.84 | 2.56 | 1.47 | 2.13 | 1.9563 | 10.079 |
| Cimetidine | 1.84 | 2.56 | 1.47 | 2.13 | 1.9563 | 10.079 |
| Doxorubicin | 3.68 | 3.49 | 1.92 | 3.44 | 3.7284 | 20.867 |
| Erythromycin | 2.9 | 3.73 | 1.25 | 4.96 | 5.773 |  |
| Mitoxantrone | 3.26 | 3.98 | 2.16 | 3.57 | 3.29 | 18.008 |
| netivudine | 2.09 | 2.64 | 1.25 | 2.1 | 1.919 | 10.751 |
| Sucrose | 2.33 | 2.86 | 2.24 | 3.06 | 2.2279 | 11.772 |
| terbutaline | 1.48 | 1.54 | 1.8 | 1.38 | 1.8377 | 8.202 |
| bupropion | 1.02 | 1.23 | 0.03 | 0.61 | 1.9406 | 7.508 |
| Carbamazepine | 2.38 | 2 | 0.42 | 0.9 | 1.8106 | 9.399 |
| Chlorpromazine | 2.2 | 1.57 | 0 | 1.01 | 2.4056 | 11.84 |
| Diazepam | 2.08 | 1.55 | 0 | 1.28 | 2.0739 | 10.48 |
| Diazepam | 2.08 | 1.55 | 0 | 1.28 | 2.0739 | 10.48 |
| Flavone | 1.82 | 1.77 | 0.12 | 0.61 | 1.6697 | 8.955 |
| guanabenz | 1.9 | 1.47 | 0.55 | 0.55 | 1.5562 | 7.87 |
| Haloperidol | 1.9 | 1.39 | 0.4 | 1.76 | 2.798 |  |
| Loxapine | 2.41 | 1.26 | 0 | 1.59 | 2.3899 | 11.519 |
| Midazolam | 2.57 | 2.01 | 0 | 1.38 | 2.2629 |  |
| progesterone | 1.45 | 3.29 | 0 | 1.14 | 2.6215 |  |
| Quetiapine | 2.86 | 1.88 | 0.25 | 2.05 | 2.9124 | 14.225 |
| Testosterone | 1.54 | 2.59 | 0.32 | 1.19 | 2.3827 |  |
| Ziprasidone | 3.62 | 2.73 | 0.38 | 1.79 | 2.9243 | 15.099 |
| Terfenadine | 2.64 | 2.64 | 0.61 | 2.09 | 4.0132 | 16.728 |
| Ritonavir | 4.08 | 4.32 | 0.87 | 3.38 | 5.5478 | 24.507 |
| acrivastine | 2.36 | 2.85 | 0.46 | 1.6 | 2.8126 | 12.148 |
| Alanine | 0.35 | 0.89 | 0.73 | 1 | 0.7055 | 2.966 |
| amoxicillin | 2.83 | 2.74 | 1.42 | 2.7 | 2.5356 | 13.171 |
| AZT | 2.87 | 2.99 | 1.3 | 2.6 | 2.7607 | 13.987 |
| bretylium |  |  |  |  |  |  |
| Cefaclor | 3 | 2.39 | 0.89 | 2.3 | 2.4154 | 12.748 |
| Cefadroxil | 3.05 | 2.6 | 1.42 | 2.48 | 2.4926 | 13.668 |
| cefatrizine | 3.94 | 3.51 | 2.32 | 2.81 | 3.0427 | 17.706 |
| Cefazolin | 3.66 | 4.22 | 0.72 | 2.5 | 2.8265 | 16.667 |
| Cefsulodine |  |  |  |  |  |  |
| Ceftazidime |  |  |  |  |  |  |
| Ceftriaxon | 4.52 | 3.47 | 1.52 | 4.12 | 3.4802 | 20.368 |
| Cephalexin | 2.87 | 2.24 | 0.89 | 2.32 | 2.4339 | 12.495 |
| Cephaloglycin | 2.94 | 2.71 | 0.89 | 2.75 | 2.7901 | 14.128 |
| Cephaloridine | |  |  |  |  |  |
| Cephradine | 2.8 | 2.11 | 0.89 | 2.36 | 2.4769 | 12.562 |
| Cetirizine | 2.25 | 2.61 | 0.46 | 2.1 | 2.9388 | 12.868 |
| gabapentin | 0.68 | 0.79 | -1.13 | 0.89 | 1.4423 | 5.696 |
| Gatifloxacin | 2.58 | 2.62 | 0.66 | 2.1 | 2.6452 | 13.361 |
| L-Dopa | 2.87 | 2.99 | 1.3 | 2.6 | 2.7607 | 13.987 |
| Leucine | 0.44 | 0.96 | 0.62 | 1 | 1.1282 | 4.291 |
| lisinopril | 1.88 | 3.22 | 1.27 | 3.15 | 3.1852 | 14.408 |
| Lomefloxacin | 2.07 | 2.48 | 0.53 | 1.89 | 2.431 | 11.851 |
| loracarbef-D | 2.58 | 2.13 | 0.89 | 2.2 | 2.3928 | 12.007 |
| Methotrexate | 3.62 | 4.32 | 2.04 | 2.98 | 3.2197 | 17.738 |
| Moxifloxacin | 2.76 | 2.9 | 0.44 | 2.1 | 2.8184 | 14.827 |
| olsalazine | 2.33 | 2.41 | 1.74 | 0.92 | 2.0288 | 11.639 |
| Phenylalanine | 1.07 | 1.44 | 0.62 | 1.17 | 1.3133 | 6.064 |
| Salicylic acid | 0.9 | 0.85 | 0.73 | 0.37 | 0.9904 | 4.732 |
| Sulfasalazine | 2.93 | 3.16 | 0.99 | 1.63 | 2.7022 | 14.625 |
| Taurocholic acid | 2.32 | 4.14 | 1.54 | 2.9 | 3.9688 | 18.542 |

# References

1. Endo S, Escher BI, Goss K-U. Capacities of Membrane Lipids to Accumulate Neutral Organic Chemicals. Environ Sci Technol. 2011;45: 5912–5921. doi:10.1021/es200855w

2. Endo S, Goss K. Applications of Polyparameter Linear Free Energy Relationships in Environmental Chemistry. Environ Sci Technol. American Chemical Society; 2014;48: 12477–12491. doi:10.1021/es503369t

3. Ulrich S.; Brown, T.N.; Watanabe, N.; Bronner, G.; Abraham, M.H.; Goss, K.-U. N. E. UFZ-LSER database v 3.2 [Internet] [Internet]. Leipzig, Deutschland, Helmholtz Zentrum für Umweltforschung - UFZ; 2017. Available: http://www.ufz.de/lserd

4. Stephens T, Quay A, Chou V. Correlation of solute transfer into alkane solvents from water and from the gas phase with updated Abraham model equations. Glob J Phys Chem. 2012;3: 1–12. Available: http://digital.library.unt.edu/ark:/67531/metadc152452/m2/1/high_res_d/Pub-561.pdf

5. Avdeef A. Leakiness and size exclusion of paracellular channels in cultured epithelial cell monolayers-interlaboratory comparison. Pharm Res. 2010;27: 480–489. doi:10.1007/s11095-009-0036-7

6. Avdeef A, Artursson P, Neuhoff S, Lazorova L, Gråsjö J, Tavelin S. Caco-2 permeability of weakly basic drugs predicted with the double-sink PAMPA pKa(flux) method. Eur J Pharm Sci. 2005;24: 333–49. doi:10.1016/j.ejps.2004.11.011

7. Kowert BA, Dang NC, Sobush KT, Seele III LG. Diffusion of Aromatic Hydrocarbons in n-Alkanes and Cyclohexanes. J Phys Chem A. 2001;105: 1232–1237. doi:10.1021/jp003002e

8. Bemporad D, Jonathan W, Luttmann C. Permeation of small molecules through a lipid bilayer: a computer simulation study. J Phys Chem B. ACS Publications; 2004;108: 4875–4884. doi:10.1021/jp035260s

9. Diamond JM, Katz Y. Interpretation of nonelectrolyte partition coefficients between dimyristoyl lecithin and water. J Membr Biol. 1974;17: 121–154. doi:10.1007/BF01870176

10. Johnson ME, Berk DA, Blankschtein D, Golan DE, Jain RK, Langer RS. Lateral diffusion of small compounds in human stratum corneum and model lipid bilayer systems. Biophys J. 1996;71: 2656–2668. doi:10.1016/S0006-3495(96)79457-2

11. Shelley JC, Calkins D, Sullivan AP. Evaluation of p K a Estimation Methods on 211 Druglike Compounds. J Chem Inf Model. 2011;51: 102–104. doi:10.1021/ci100332m

12. Garberg P, Ball M, Borg N, Cecchelli R, Fenart L, Hurst RD, et al. In vitro models for the blood–brain barrier. Toxicol Vitr. 2005;19: 299–334. doi:10.1016/j.tiv.2004.06.011

13. von Richter O, Glavinas H, Krajcsi P, Liehner S, Siewert B, Zech K. A novel screening strategy to identify ABCB1 substrates and inhibitors. Naunyn Schmiedebergs Arch Pharmacol. 2009;379: 11–26. doi:10.1007/s00210-008-0345-0

14. Liang E, Chessic K, Yazdanian M. Evaluation of an accelerated Caco‐2 cell permeability model. J Pharm Sci. 2000;89: 336–345. doi:10.1002/(SICI)1520-6017(200003)89:3<336::AID-JPS5>3.0.CO;2-M

15. Adson A, Raub TJ, Burton PS, Barsuhn CL, Hilgers a R, Audus KL, et al. Quantitative approaches to delineate paracellular diffusion in cultured epithelial cell monolayers. J Pharm Sci. 1994;83: 1529–36. doi:10.1002/jps.2600831103

16. Mueller P, Rudin DO, Tien HT, Wescott WC. METHODS FOR THE FORMATION OF SINGLE BIMOLECULAR LIPID MEMBRANES IN AQUEOUS SOLUTION. J Phys Chem. 1963;67: 534–535. doi:10.1021/j100796a529

17. Walter A, Gutknecht J. Permeability of small nonelectrolytes through lipid bilayer membranes. J Membr Biol. 1986;90: 207–217. Available: http://www.ncbi.nlm.nih.gov/pubmed/3735402

18. Gutknecht J, Tosteson DC. Diffusion of Weak Acids across Lipid Bilayer Membranes: Effects of Chemical Reactions in the Unstirred Layers. Science (80- ). 1973;182: 1258–1261. doi:10.1126/science.182.4118.1258

19. Walter A, Hastings D, Gutknecht J. Weak acid permeability through lipid bilayer membranes. Role of chemical reactions in the unstirred layer. J Gen Physiol. 1982;79: 917–933. doi:10.1085/jgp.79.5.917

20. Antonenko YN, Yaguzhinsky LS. The role of pH gradient in the unstirred layers in the transport of weak acids and bases through bilayer lipid membranes. Bioelectrochemistry Bioenerg. 1984;13: 85–91. doi:10.1016/0302-4598(84)85117-X

21. Antonenko YN, Denisov GA, Pohl P. Weak acid transport across bilayer lipid membrane in the presence of buffers. Theoretical and experimental pH profiles in the unstirred layers. Biophys J. 1993;64: 1701–1710. doi:10.1016/S0006-3495(93)81542-X

22. Finkelstein A. Water and nonelectrolyte permeability of lipid bilayer membranes. J Gen Physiol. 1976;68: 127–135. doi:10.1085/jgp.68.2.127

23. Orbach E, Finkelstein A. The nonelectrolyte permeability of planar lipid bilayer membranes. J Gen Physiol. 1980;75: 427–436.

24. Xiang TX, Anderson BD. The relationship between permeant size and permeability in lipid bilayer membranes. J Membr Biol. 1994;140: 111–122. doi:10.1007/BF00232899

25. Xiang TX, Chen X, Anderson BD. Transport methods for probing the barrier domain of lipid bilayer membranes. Biophys J. 1992;63: 78–88. doi:10.1016/S0006-3495(92)81581-3

26. Walter a, Gutknecht J. Monocarboxylic acid permeation through lipid bilayer membranes. J Membr Biol. 1984;77: 255–264. doi:10.1007/BF01870573

27. Xiang TX, Anderson BD. Substituent contributions to the transport of substituted p-toluic acids across lipid bilayer membranes. J Pharm Sci. 1994;83: 1511–1518. doi:10.1002/jps.2600831027

28. LeBlanc OH. The effect of uncouplers of oxidative phosphorylation on lipid bilayer membranes: Carbonylcyanide m-chlorophenylhydrazone. J Membr Biol. 1971;4: 227–251. doi:10.1007/BF02431973

29. Gutknecht J, Walter A. Histamine, theophylline and tryptamine transport through lipid bilayer membranes. Biochim Biophys Acta - Biomembr. 1981;649: 149–154. doi:10.1016/0005-2736(81)90401-6

30. Gutknecht J, Walter A. Hydrofluoric and nitric acid transport through lipid bilayer membranes. Biochimica et Biophysica Acta (BBA) - Biomembranes. 1981. pp. 153–156. doi:10.1016/0005-2736(81)90071-7

31. Pohl P, Rokitskaya TI, Pohl EE, Saparov SM. Permeation of phloretin across bilayer lipid membranes monitored by dipole potential and microelectrode measurements. Biochim Biophys Acta - Biomembr. 1997;1323: 163–172. doi:10.1016/S0005-2736(96)00185-X

32. Saparov SM, Antonenko YN, Pohl P. A New Model of Weak Acid Permeation through Membranes Revisited: Does Overton Still Rule? Biophys J. Elsevier; 2006;90: L86–L88. doi:10.1529/biophysj.106.084343

33. Zocher F, van der Spoel D, Pohl P, Hub JS. Local Partition Coefficients Govern Solute Permeability of Cholesterol-Containing Membranes. Biophys J. Biophysical Society; 2013;105: 2760–2770. doi:10.1016/j.bpj.2013.11.003

34. Gutknecht J, Walter A. SCN− and HSCN transport through lipid bilayer membranes. Biochim Biophys Acta - Biomembr. 1982;685: 233–240. doi:10.1016/0005-2736(82)90062-1

35. Irvine JD, Takahashi L, Lockhart K, Cheong J, Tolan JW, Selick HE, et al. MDCK (Madin-Darby canine kidney) cells: A tool for membrane permeability screening. J Pharm Sci. 1999;88: 28–33. doi:10.1021/js9803205

36. Robertson SM, Curtis MA, Schlech BA, Rusinko A, Owen GR, Dembinska O, et al. Ocular Pharmacokinetics of Moxifloxacin After Topical Treatment of Animals and Humans. Surv Ophthalmol. 2005;50: S32–S45. doi:10.1016/j.survophthal.2005.07.001

37. Hayeshi R, Masimirembwa C, Mukanganyama S, Ungell A-LB. The potential inhibitory effect of antiparasitic drugs and natural products on P-glycoprotein mediated efflux. Eur J Pharm Sci. 2006;29: 70–81. doi:10.1016/j.ejps.2006.05.009

38. Summerfield SG, Read K, Begley DJ, Obradovic T, Hidalgo IJ, Coggon S, et al. Central Nervous System Drug Disposition: The Relationship between in Situ Brain Permeability and Brain Free Fraction. J Pharmacol Exp Ther. 2007;322: 205–213. doi:10.1124/jpet.107.121525

39. Obradovic T, Dobson GG, Shingaki T, Kungu T, Hidalgo IJ. Assessment of the first and second generation antihistamines brain penetration and role of P-glycoprotein. Pharm Res. 2007;24: 318–327. doi:10.1007/s11095-006-9149-4

40. Alsenz J, Haenel E. Development of a 7-day, 96-well Caco-2 permeability assay with high-throughput direct UV compound analysis. Pharm Res. 2003;20: 1961–1969. doi:10.1023/B:PHAM.0000008043.71001.43

41. Raeissi SD, Li J, Hidalgo IJ. The Role of an α-Amino Group on H + -dependent Transepithelial Transport of Cephalosporins in Caco-2 Cells. J Pharm Pharmacol. 1999;51: 35–40. doi:10.1211/0022357991772060

42. Wang Q, Rager JD, Weinstein K, Kardos PS, Dobson GL, Li J, et al. Evaluation of the MDR-MDCK cell line as a permeability screen for the blood–brain barrier. Int J Pharm. 2005;288: 349–359. doi:10.1016/j.ijpharm.2004.10.007

43. Thiel-Demby VE, Humphreys JE, St. John Williams LA, Ellens HM, Shah N, Ayrton AD, et al. Biopharmaceutics Classification System: Validation and Learnings of an in Vitro Permeability Assay. Mol Pharm. 2009;6: 11–18. doi:10.1021/mp800122b
